# Supplementary material for: Dual Targeting Oncoproteins MYC and HIF1α Regresses Tumor Growth of Lung Cancer and Lymphoma
Source: Cancers (Basel). 2021 Feb 9;13(4):694. doi: 10.3390/cancers13040694 (PMC7914643; doi:10.3390/cancers13040694)
Supplement: Supplementary file 1 [file cancers-13-00694-s001.pdf]

cancers MDPI

Supplementary Materials: Dual Targeting Oncoproteins MYC and HIF1α Regresses Tumor Growth of Lung Cancer and Lymphoma

Xiaohu Huang, Yan Liu, Yin Wang, Christopher Bailey, Pan Zheng and Yang Liu

Table S1. qRT-PCR primers.

	Name	Sequence
Primers for human	hc-Myc-qPCR-F1	CCTACCCTCTCAACGACAGC
	hc-Myc-qPCR-R1	TTCCTCCTCAGAGTCGCTGC
	hc-Myc-qPCR-F2	TGCCCATTTGGGGACACTTC
	hc-Myc-qPCR-R2	GGGAGGCTGCTGGTTTTCCA
	hc-Myc-qPCR-F3	TGAGGAGACACCGCCCAC
	hc-Myc-qPCR-R3	CAACATCGATTTCTTCCTCATCTTC
	hc-Myc-qPCR-F4	AATGAAAAGGCCCCCAAGGTAG-TTATCC
	hc-Myc-qPCR-R4	GTCGTTTCCG-CAACAAGTCCTCTTC
	hHif1a-qPCR-F1	TGCTTGGTGCTGATTTGTGA
	hHif1a-qPCR-R1	GGTCAGATGATCAGAGTCCA
	hHif1a-qPCR-F2	GATCACCCTCTTCGTCGCTT
	hHif1a-qPCR-R2	AACTTATCTTTTTCTTGTCGTTCGC
Primers for mouse	Ms-Myc-qPCR-F1	CAGCGACTCTGAAGAAGAGCA
	Ms-Myc-qPCR-R1	TTGTGCTGGTGAGTGGAGAC
	Ms-Hif1a-qPCR-F3	AGGATGAGTTCTGAACGTCGAAA
	Ms-Hif1a-qPCR-R3	AAACCATGTCGCCGTCATCT

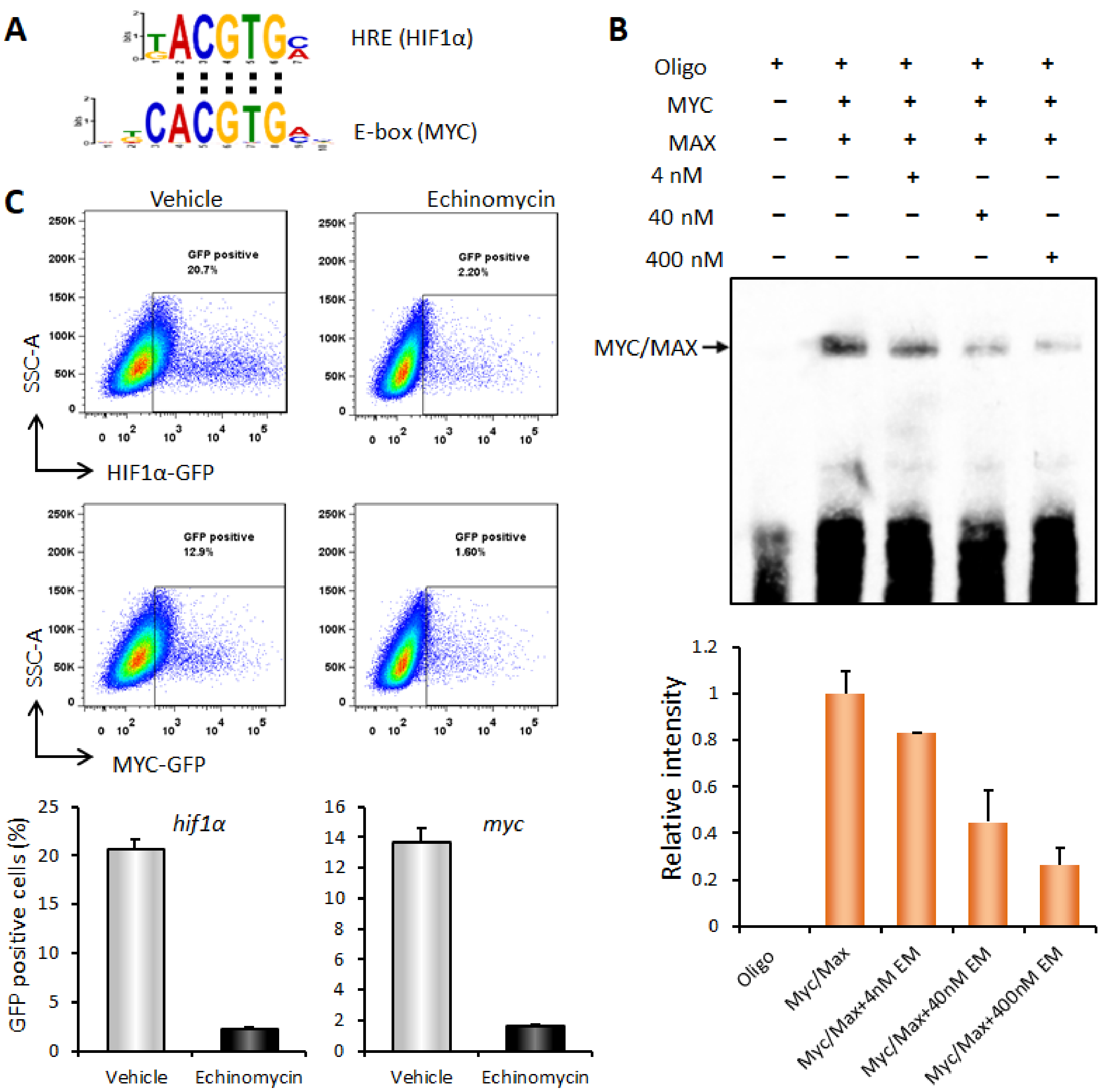

Figure S1. Inhibition of DNA binding capacity of MYC and HIF1α by echinomycin. (**A**) Sequence logos and alignment of HRE and E-box. (**B**) Electrophoretic mobility shift assay. E-box DNA binding capacity of recombinant MYC/Max complex was determined in the presence of increasing concentrations of echinomycin as indicated. (**C**) MYC and HIF1α reporter activities in HEK293 cells. HEK293 cells were transiently transfected with cDNA encoding mutant HIF1α (P402A / P564A) in conjunction with HRE-driven EGFP reporter or *myc* cDNA plus E-box-driven EGFP reporter. Then, GFP positive cells were analyzed by flow cytometry 24 hours after transduction. Experiments were repeated two times.

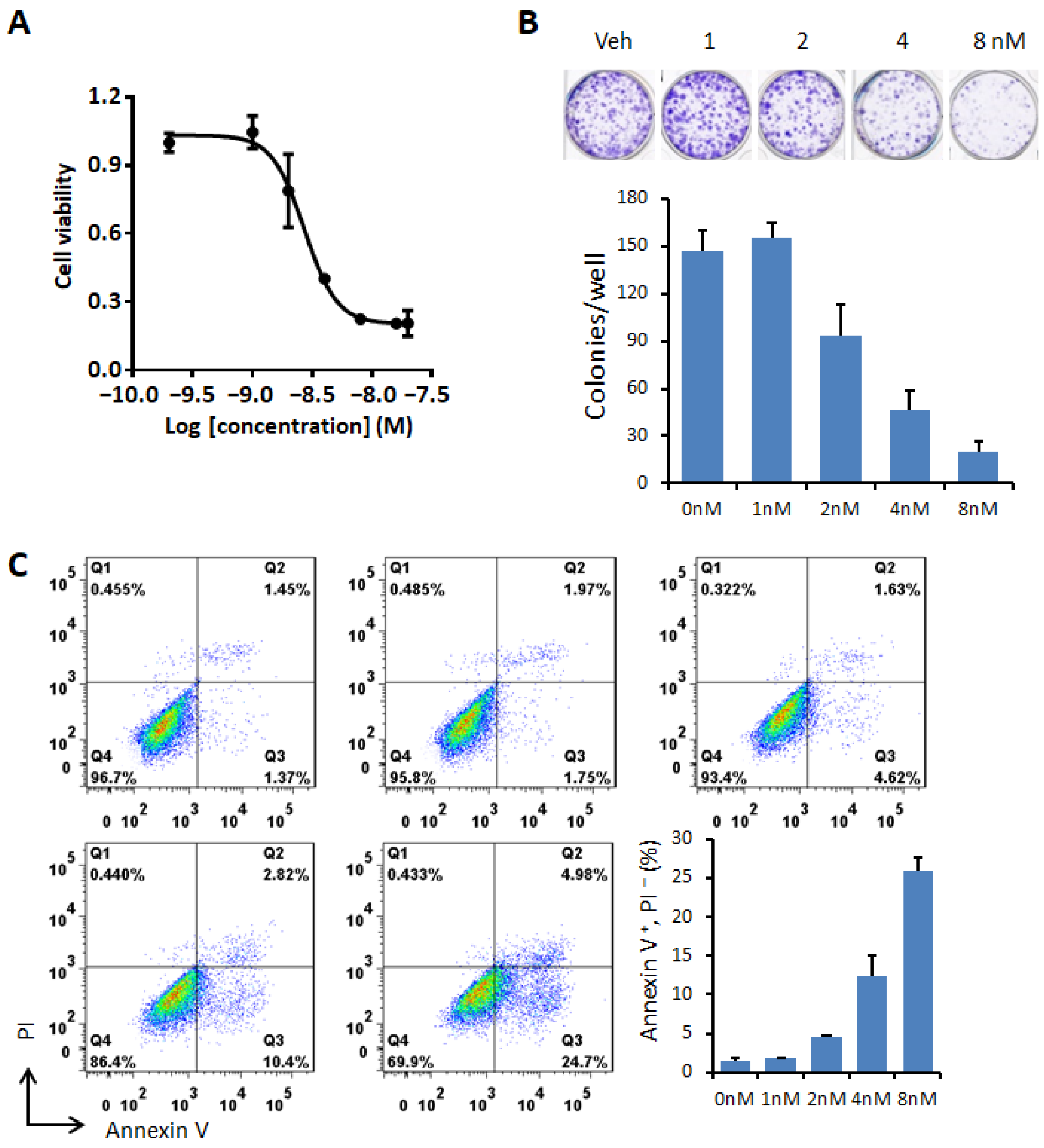

Figure S2. Inhibition of cell growth and induction of apoptosis of lung cancer cells by echinomycin. (**A**) Cell viability assay. Dose response curve of cell viability was measured by MTT assay when H1944 cells were treated with vehicle or echinomycin at different concentrations for 48 h. Data represent mean ± SEM of 4 biologic replicates. (**B**) Clonogenic assay. H1944 cells were seeded and cultured in six-well plates followed by the treatment of vehicle or different concentrations of echinomycin as indicated for 3 days. Colonies were photographed and counted. (**C**) H1944 cells were treated with different concentrations of echinomycin for 48 h, then apoptotic cells were stained with Annexin V and PI and quantified by FACS.

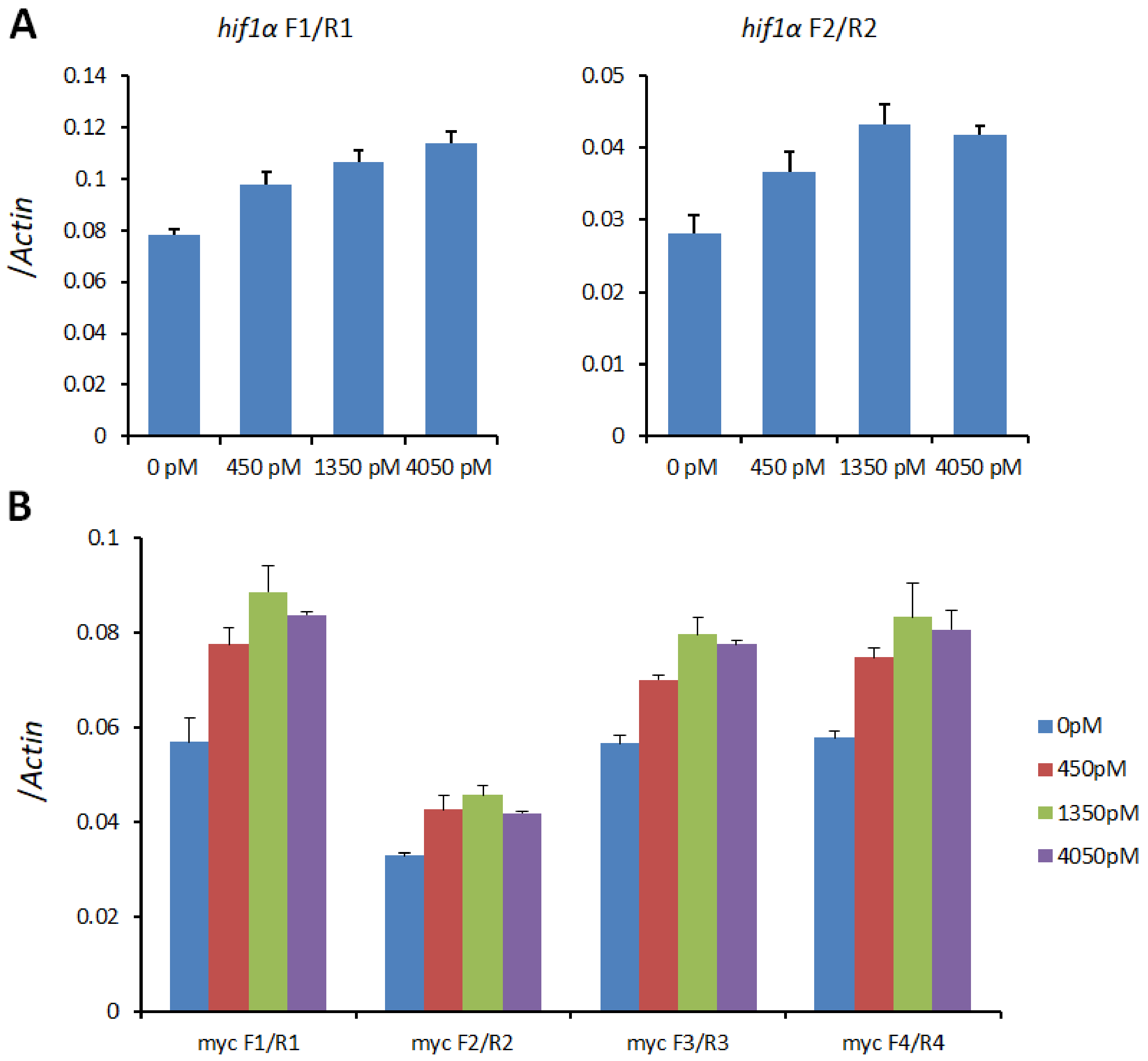

Figure S3. Echinomycin increased mRNA level of *myc* and *hif1α* in H1944 cells. (**A**) *hif1α* mRNAs in H1944 cells treated with echinomycin was quantified by qRT-PCR using two different pairs of primers spanning exon-exon junctions. Experiments were repeated four times. (**B**) *myc* mRNAs in H1944 cells treated with different concentrations of echinomycin was quantified by qRT-PCR using four different pairs of primers spanning exon-exon junctions. Experiments were repeated two times.

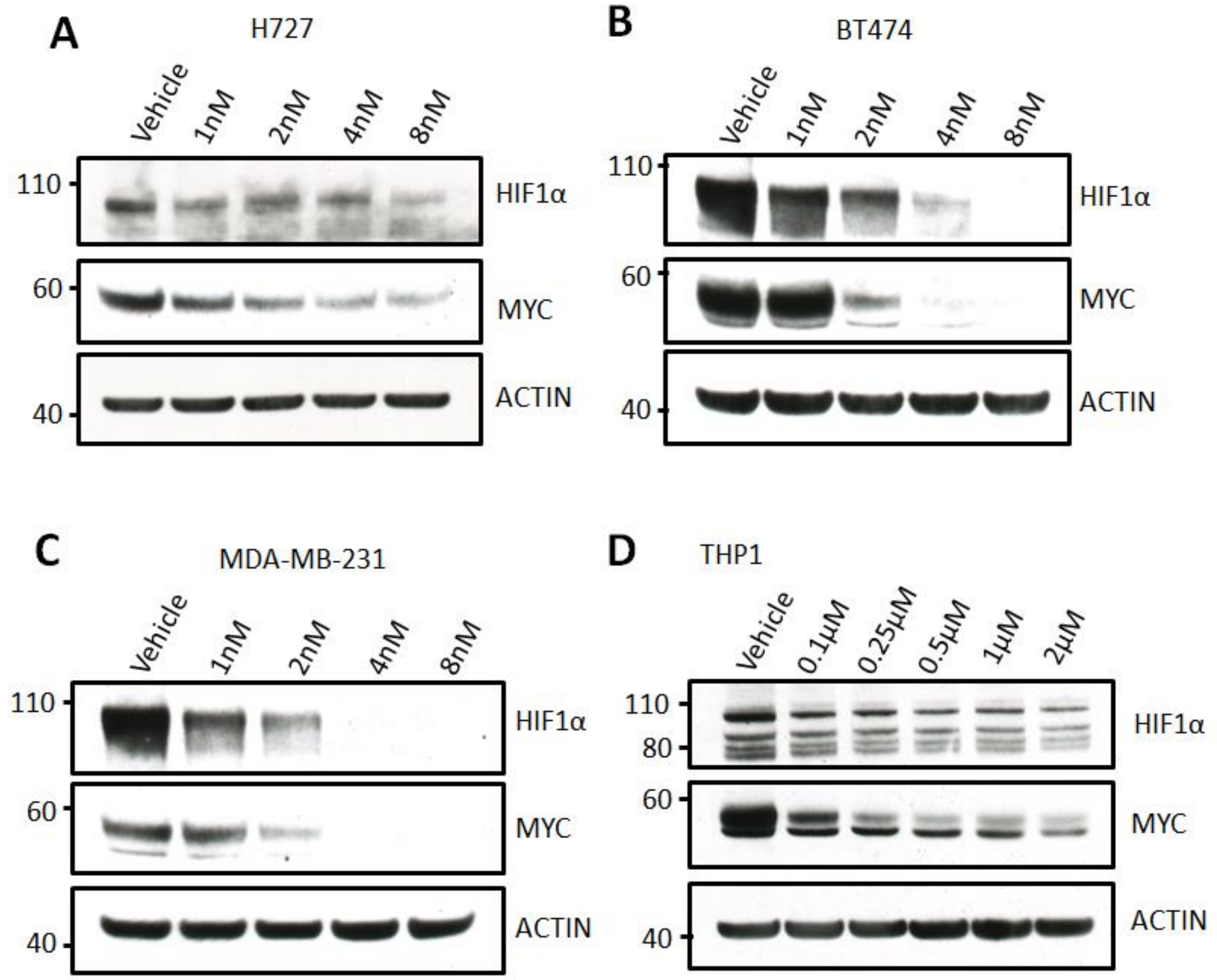

Figure S4. Induced degradation of MYC and HIF1α protein by echinomycin in lung, breast cancer and leukemia cells. MYC and HIF1α protein was checked by western blot in a non-small cell lung carcinoma cell (H727) (**A**), two breast cancer cells BT474 (**B**) and MDA-MB-231 (**C**), and leukemia cells THP1 (**D**) after treatment by echinomycin. Experiments were repeated two times.

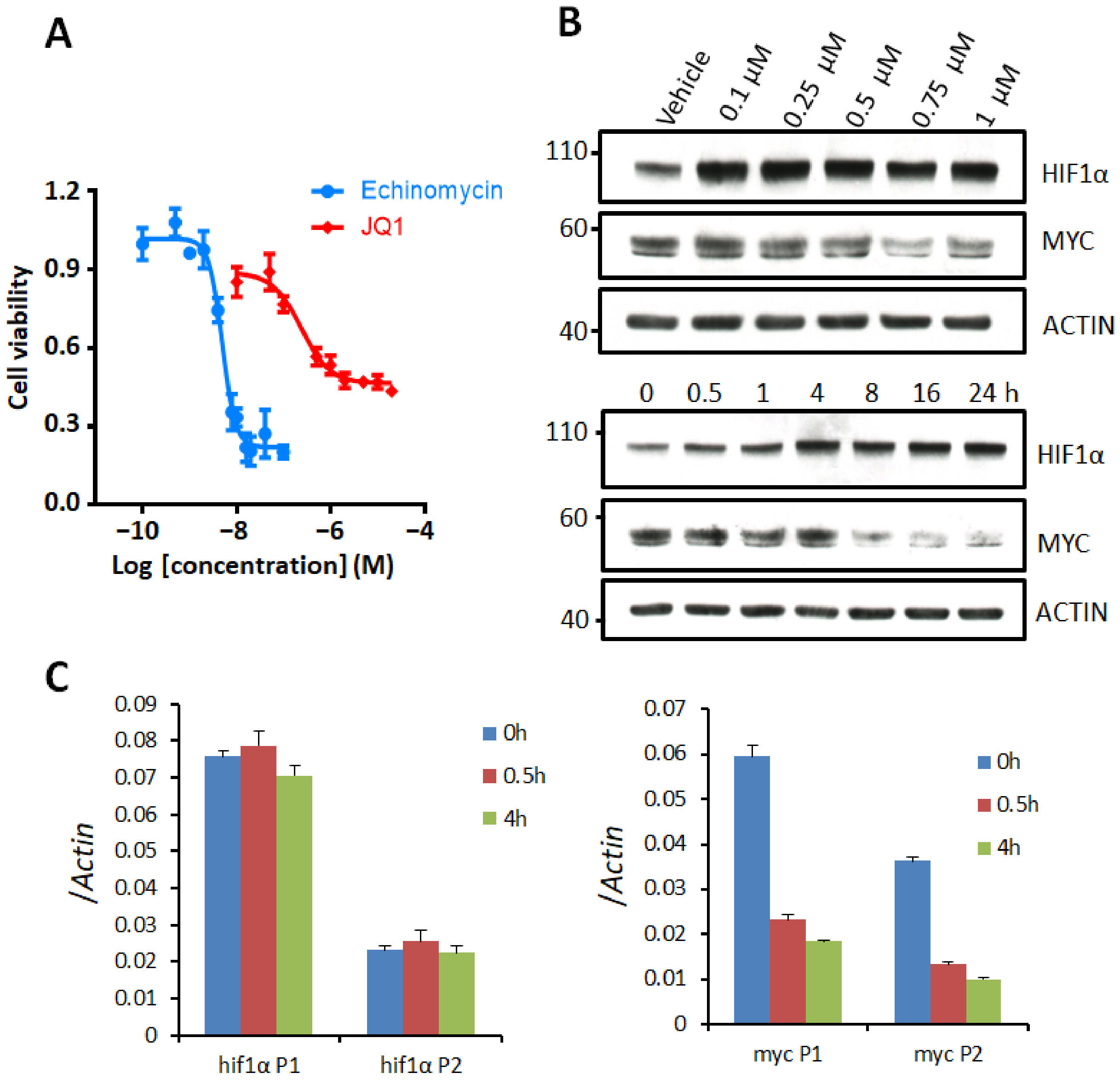

Figure S5. Effect of bromodomain inhibitor JQ1 on MYC and HIF1*α* in H1944 cells. (**A**) H1944 cells were treated with different concentrations of echinomycin or JQ1 as indicated for 48 h and then cell viability was analyzed by MTT assay ($n = 3$ replicates). (**B**) Dose- and time course-dependent change of MYC and HIF1*α* protein after JQ1 treatment for 24 h. (**C**) *myc* and *hif1α* mRNA level in H1944 cells after JQ1 treatment for 0.5 h or 4 h was quantified by qRT-PCR using two different pairs of primers, separately.

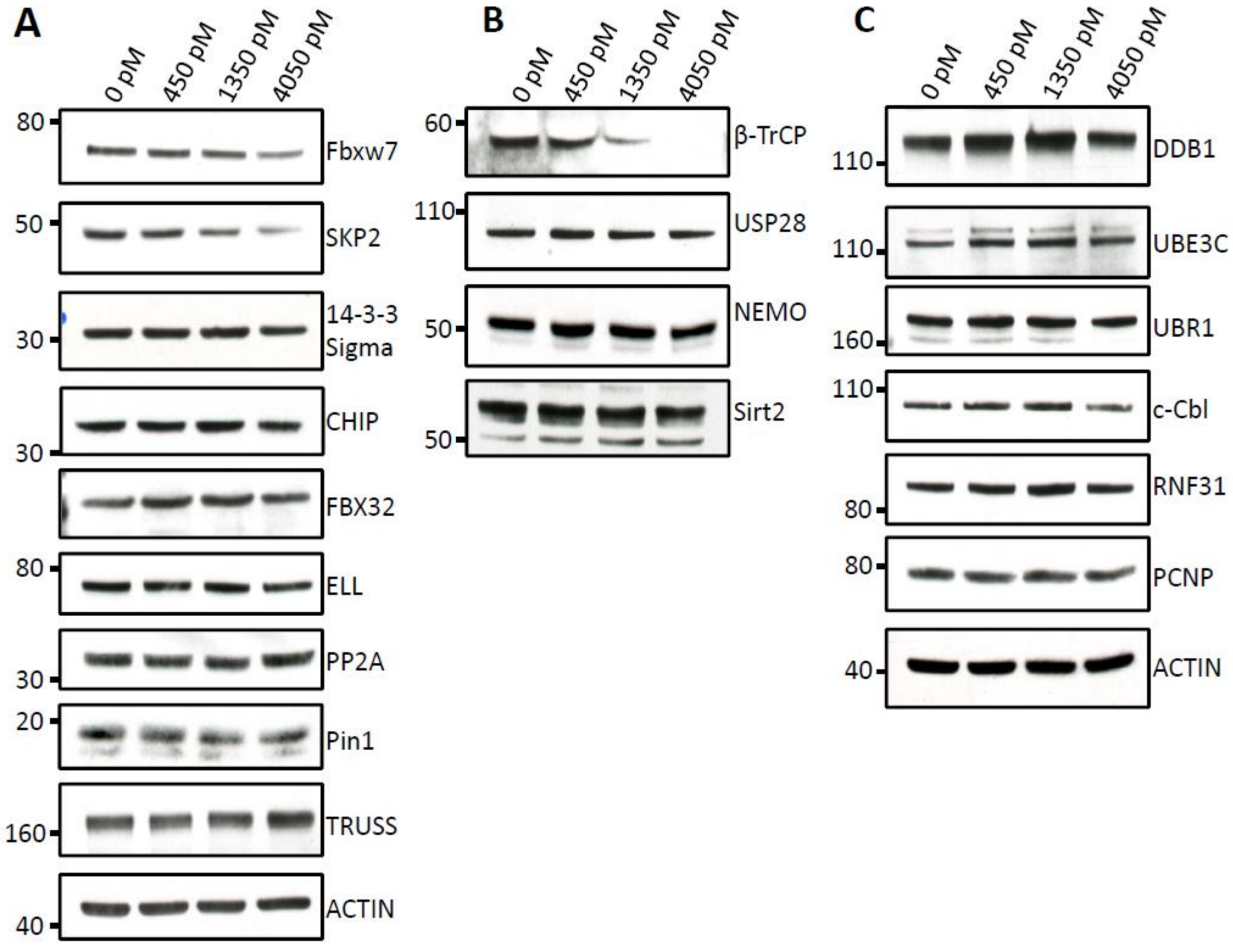

Figure S6. Western blot analysis of proteins involved in the regulation of protein stability. Western blot analysis of proteins which can mediate the degradation of MYC (**A**), enhance the stability of MYC (B) and of other known nuclear E3 ligases (**C**) in H1944 cells treated with vehicle or echinomycin at indicated concentrations for 24 h. Experiments were repeated 2–3 times.

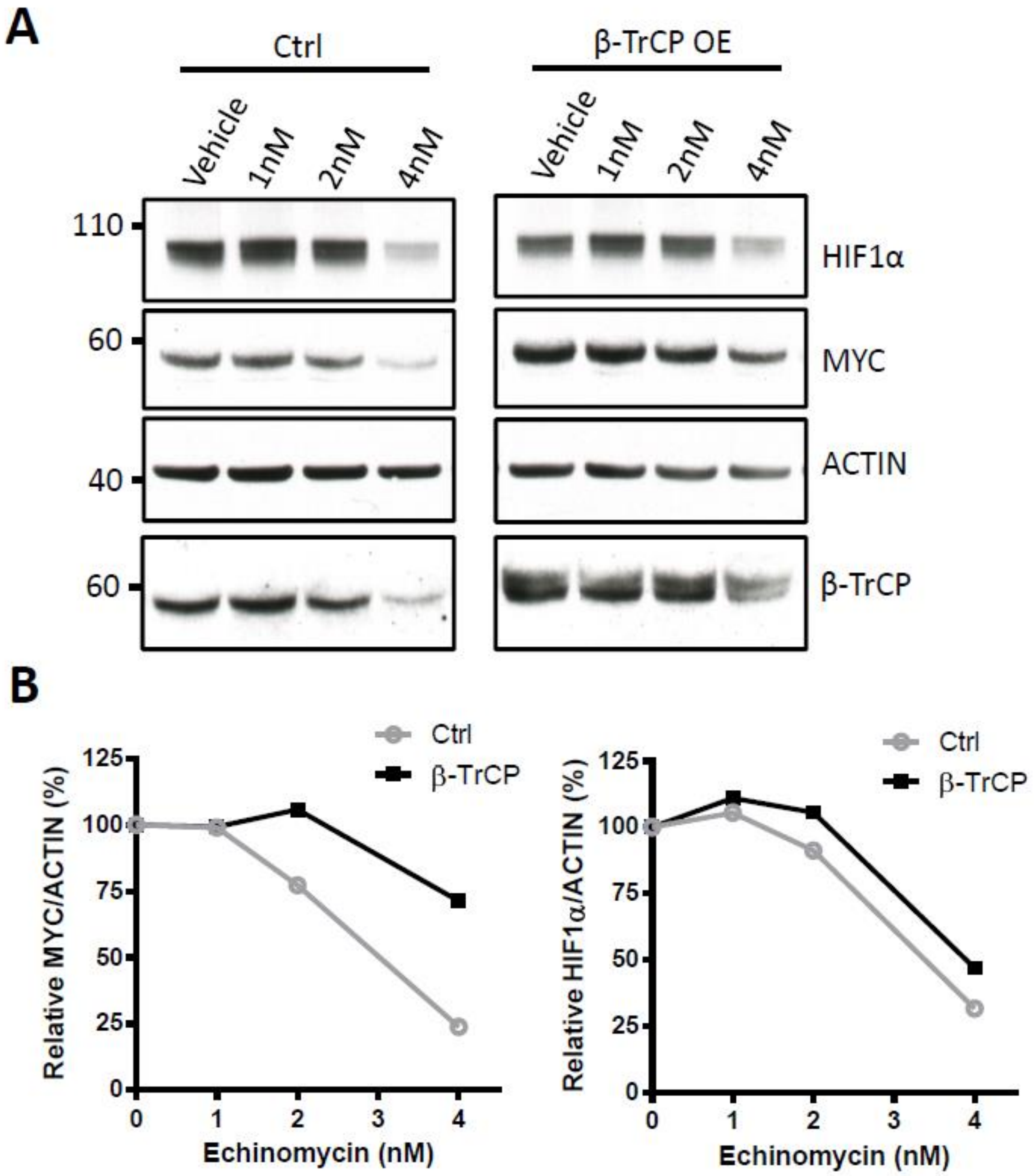

Figure S7. Response to echinomycin of β-TrCP overexpressed H1944 cells. (**A**) Western blot of MYC and β-TrCP in wild type and β-TrCP transduced H1944 cells treated with vehicle or echinomycin at indicated concentrations for 24 h. (**B**) Quantification of MYC and HIF1α in (*A*).

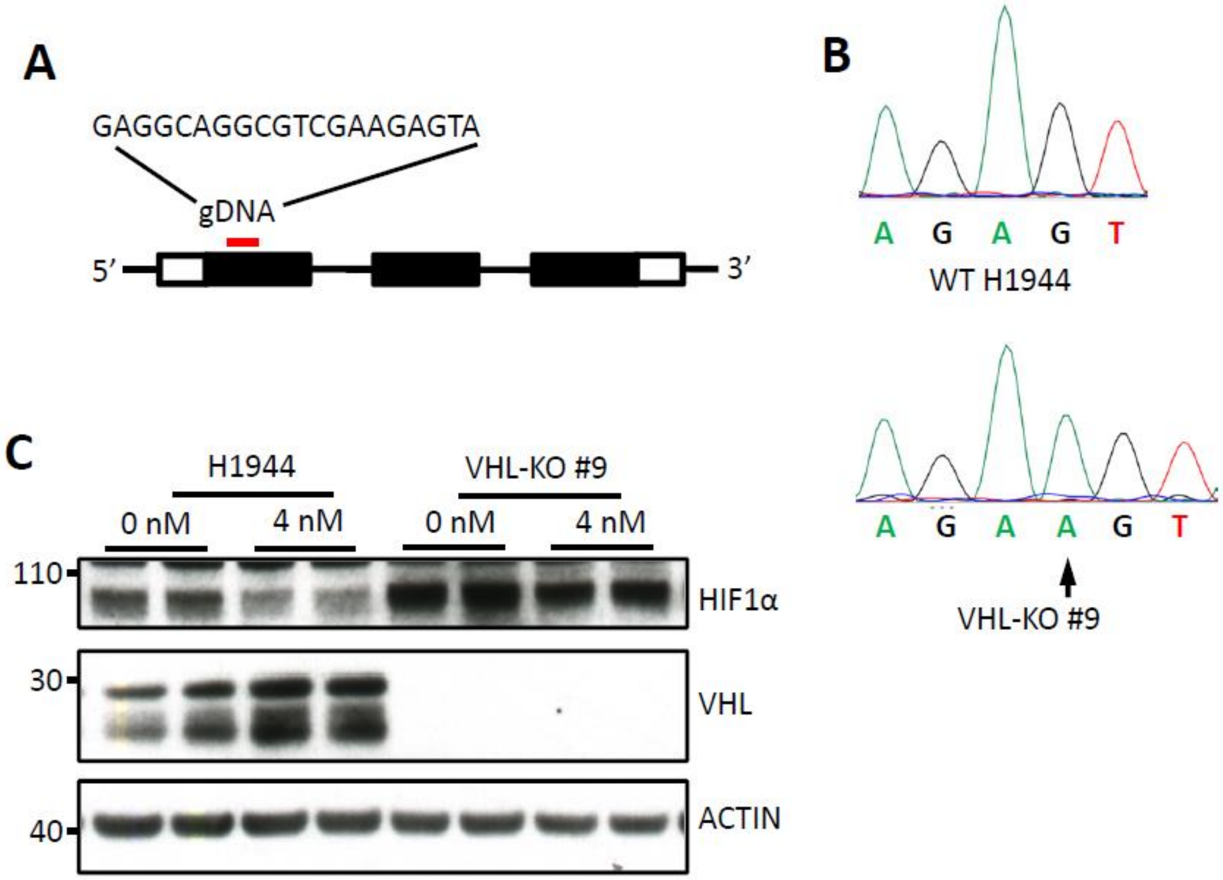

Figure S8. *vhl* CRISPR in H1944 cells. (**A**) Schematic location and sequence of guide DNA for VHL CRISPR used in H1944 cells. (**B**) Genomic DNA sequencing results show the nucleotide "A" insertion (indicated by arrow) in exon 1 of *vhl* in two clones by CRISPR. (C) Western blot analysis of HIF1α in wild type H1944 cells and VHL deficient H1944 cells (clone #9) treated with vehicle or 4 nM echinomycin for 24 h.

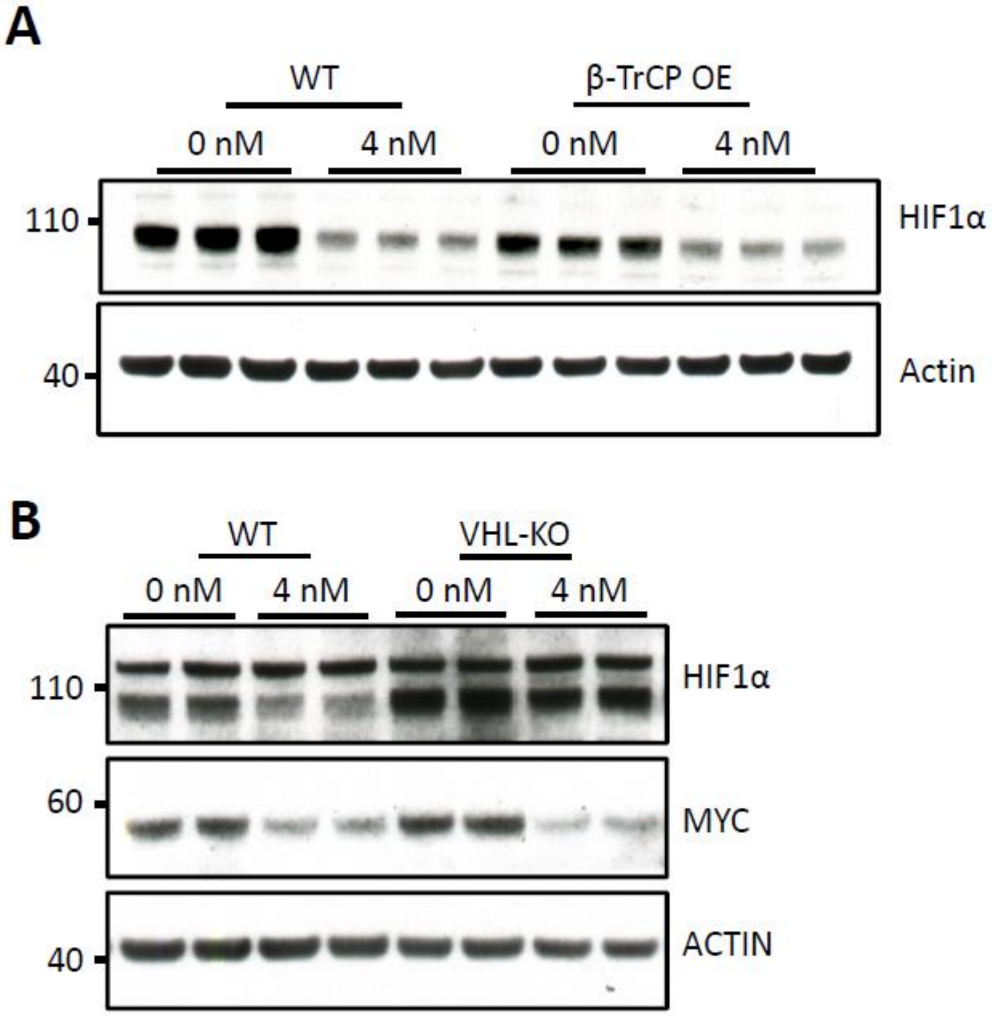

Figure S9. The two signaling pathways controlling the degradation of MYC and HIF1α are independent. (A) HIF1α protein was checked by western blot in both wild type (WT) and β-TrCP overexpressed (β-TrCP OE) H1944 cells when treated with vehicle or 4 nM echinomycin for 24 h. Note: Because this result was obtained at the same time with the results in Figure 3C, the loading control ACTIN are the same as in Figure 3C. (B) MYC and HIF1α protein was checked by western blot in both wild type (WT) and VHL knockout (VHL-KO) H1944 cells when treated with vehicle or 4 nM echinomycin for 24 h. Experiments were repeated 2 times.

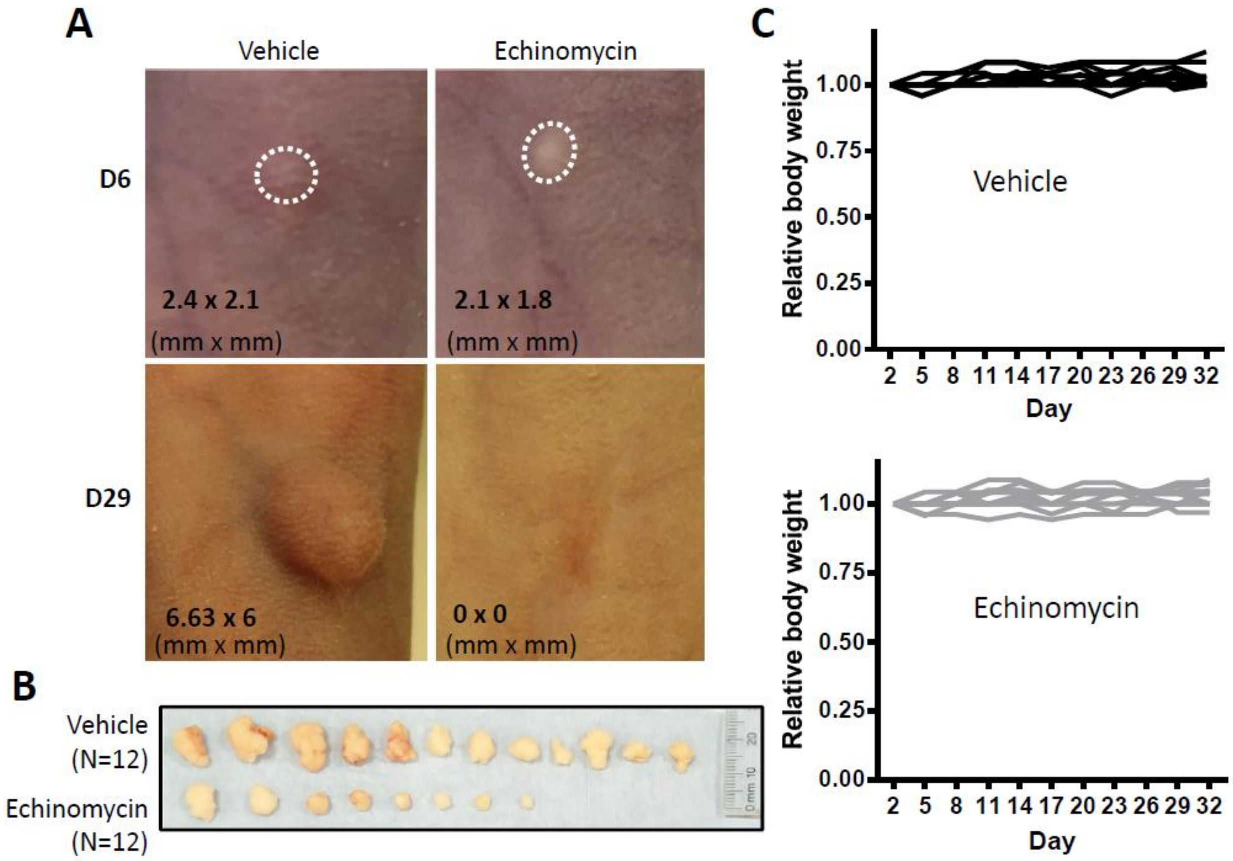

Figure S10. H1944 lung adenocarcinoma xenograft model and toxicity analysis of echinomycin on mice *in vivo*. (A) Representative images showed the tumor morphology on day 6 and day 29 after inoculation in vehicle and echinomycin treated mice. (B) Photographs of all tumors that developed in the nude mice treated with vehicle or echinomycin are presented. (C) Body weight of nude mice bearing xenogeneic human lung cancer was checked every three days in vehicle and echinomycin treated groups.

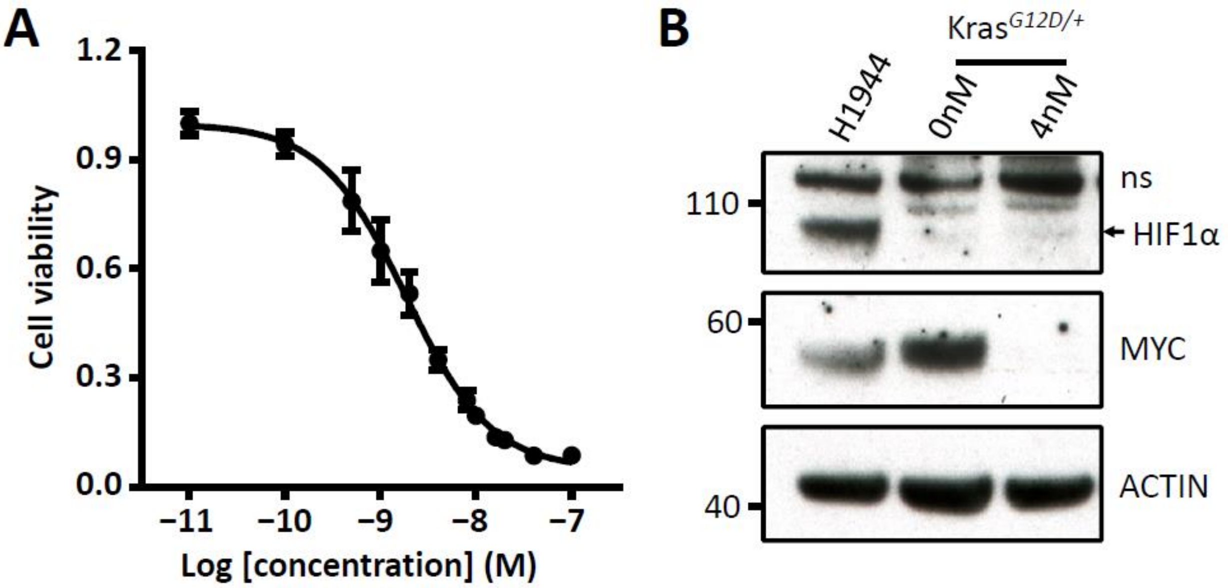

Figure S11. Response of mouse *Kras*$^{G12D/+}$; *p53*$^{-/-}$ lung cancer cells to echinomycin. (**A**) Dose response curve of mouse *Kras*$^{G12D/+}$; *p53*$^{-/-}$ lung cancer cells was measured by MTT assay when cells were treated with vehicle or echinomycin at

different concentrations for 48 h (n = 3 replicates). (**B**) Expression of MYC and HIF1α protein was determined by western blot in mouse *Kras*$^{G12D/+}$; *p53*$^{-/-}$ lung cancer cells treated with vehicle or echinomycin for 48 h.

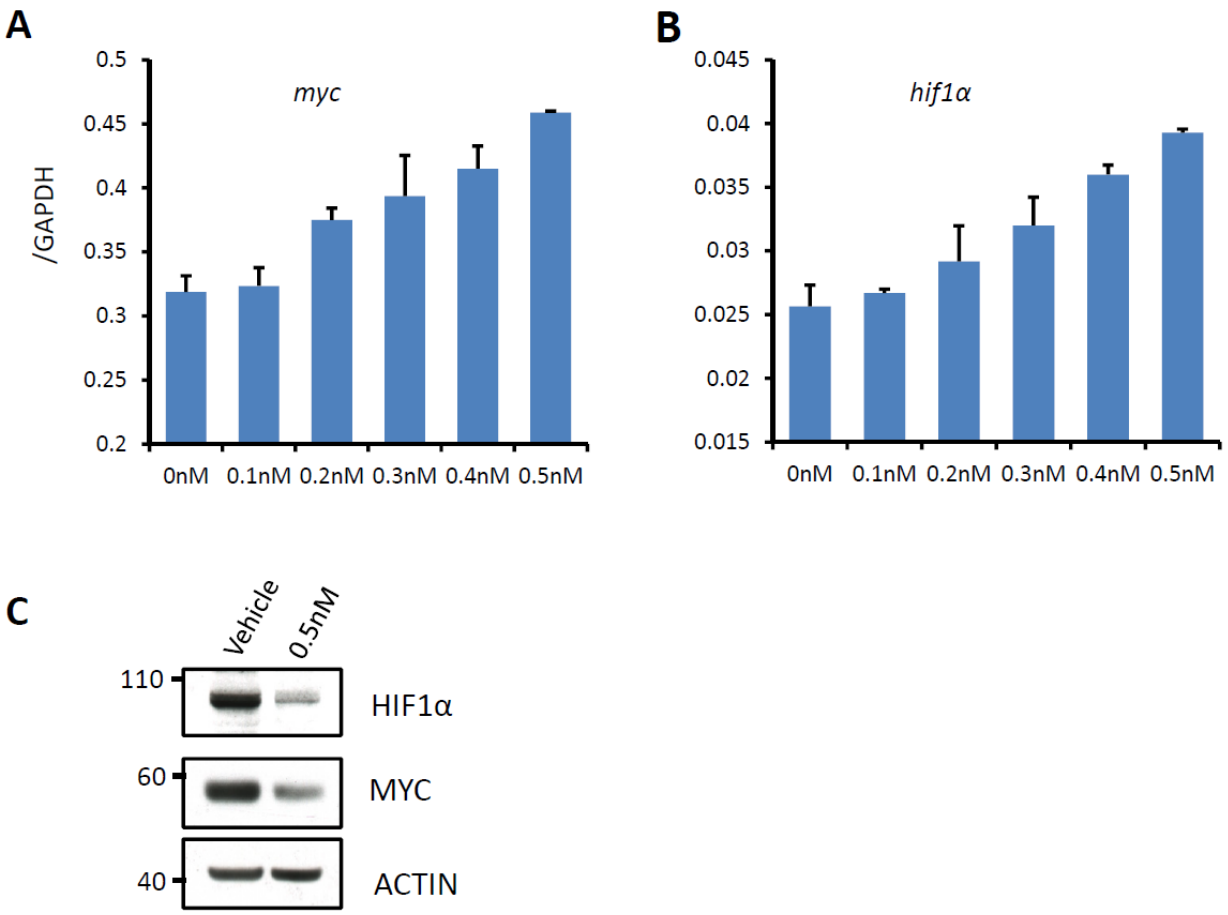

Figure S12. Effect of echinomycin on MYC and HIF1α in Eμ-Myc lymphoma cells. mRNAs of *myc* (**A**) and *hif1α* (**B**) in Eμ-Myc lymphoma cells treated with echinomycin for 24 h were determined by qRT-PCR. *GAPDH* was used as internal control. (n = 3 replicates). (C) MYC and HIF1α protein was determined by western blot in Eμ-Myc lymphoma cells treated with vehicle or echinomycin for 24 h at 0.5 nM.

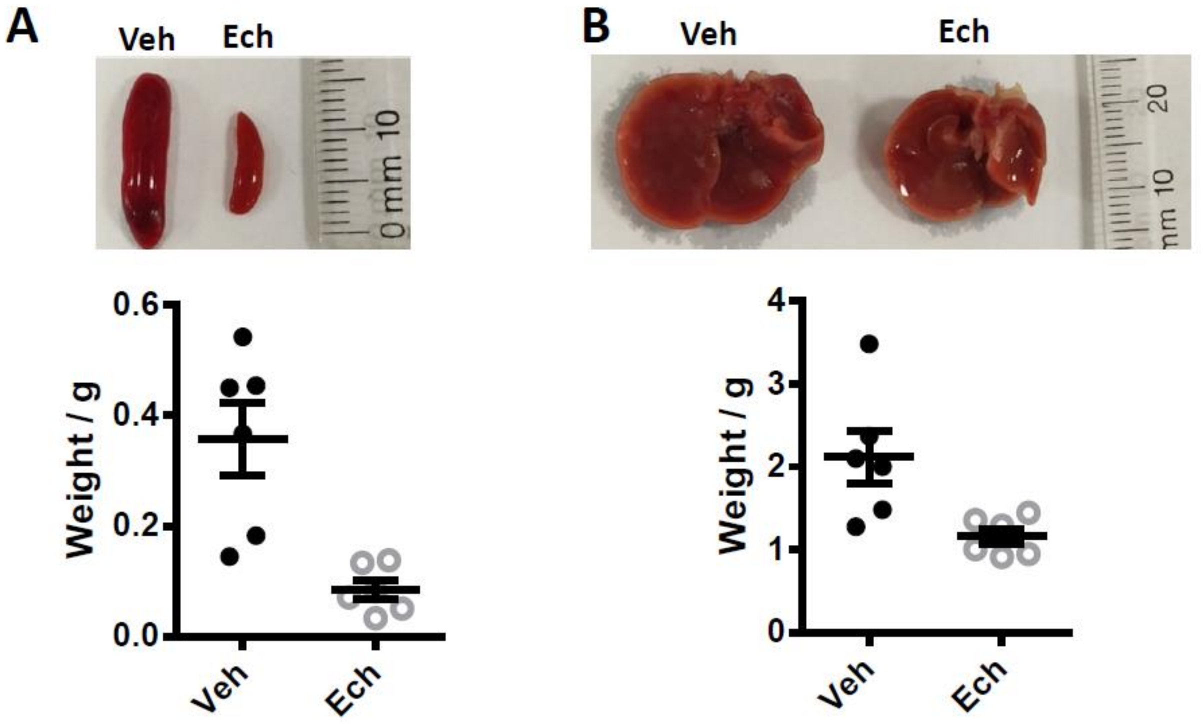

Figure S13. Morphologies of spleen and liver in Eμ-Myc lymphoma mice. (**A**) Size (upper) and weight (lower) of spleen in Eμ-Myc lymphoma mice treated with vehicle or echinomycin. (**B**) Size (upper) and weight (lower) of liver in Eμ-Myc lymphoma mice treated with vehicle or echinomycin. *n* = 6 mice / group.

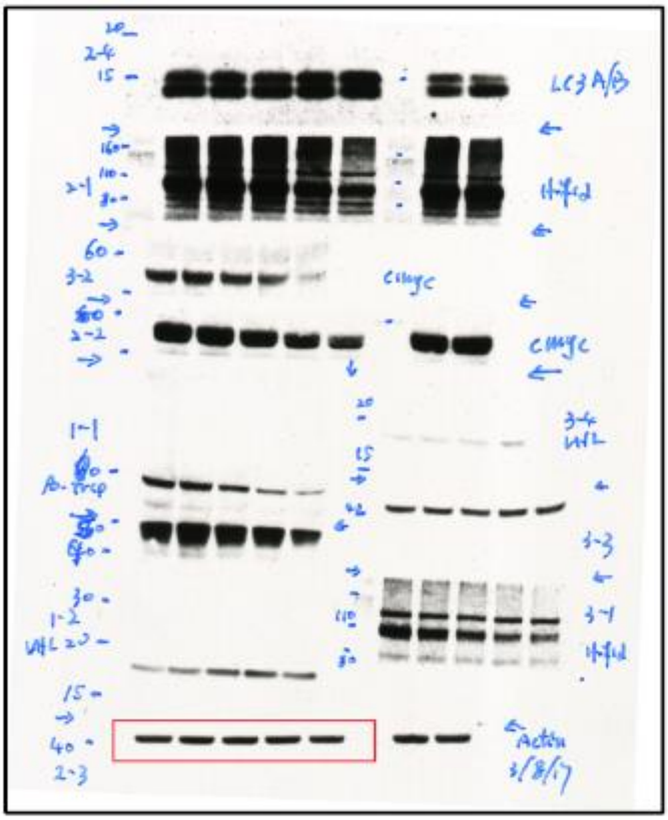

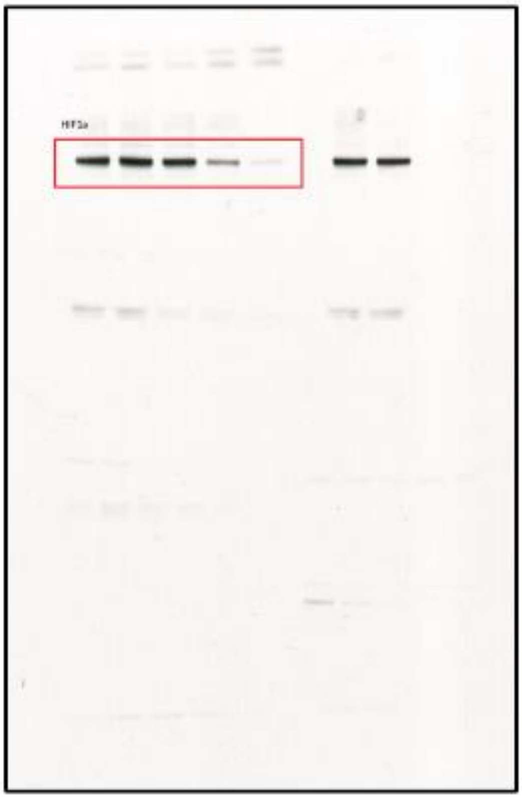

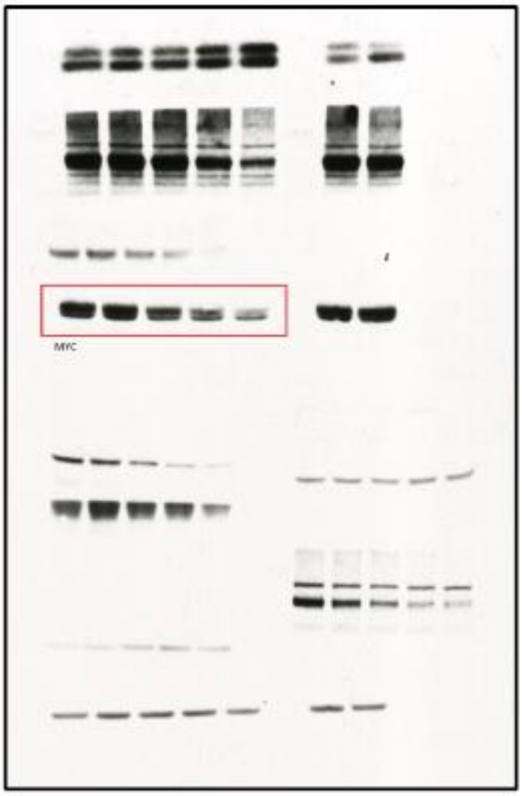
MYC

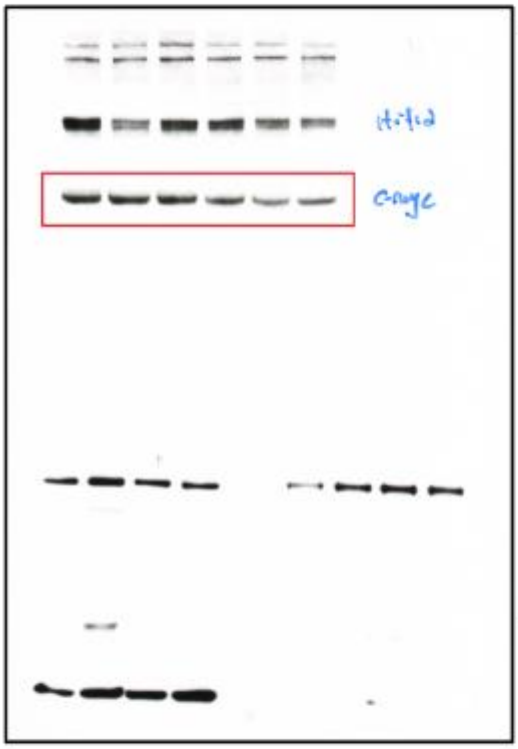
C-myc

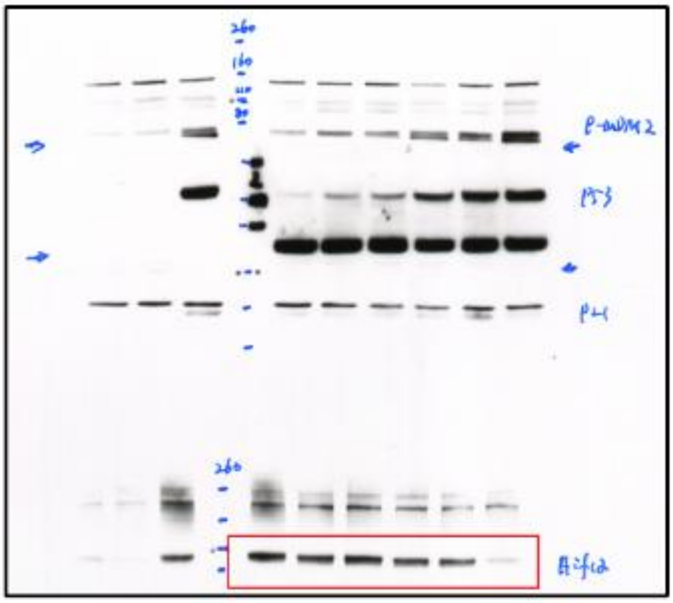
P-MDM2
P53
P21

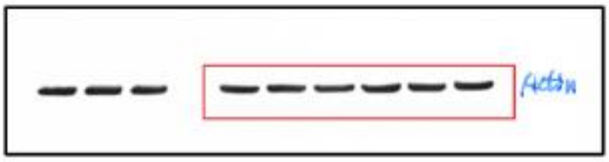
Actin

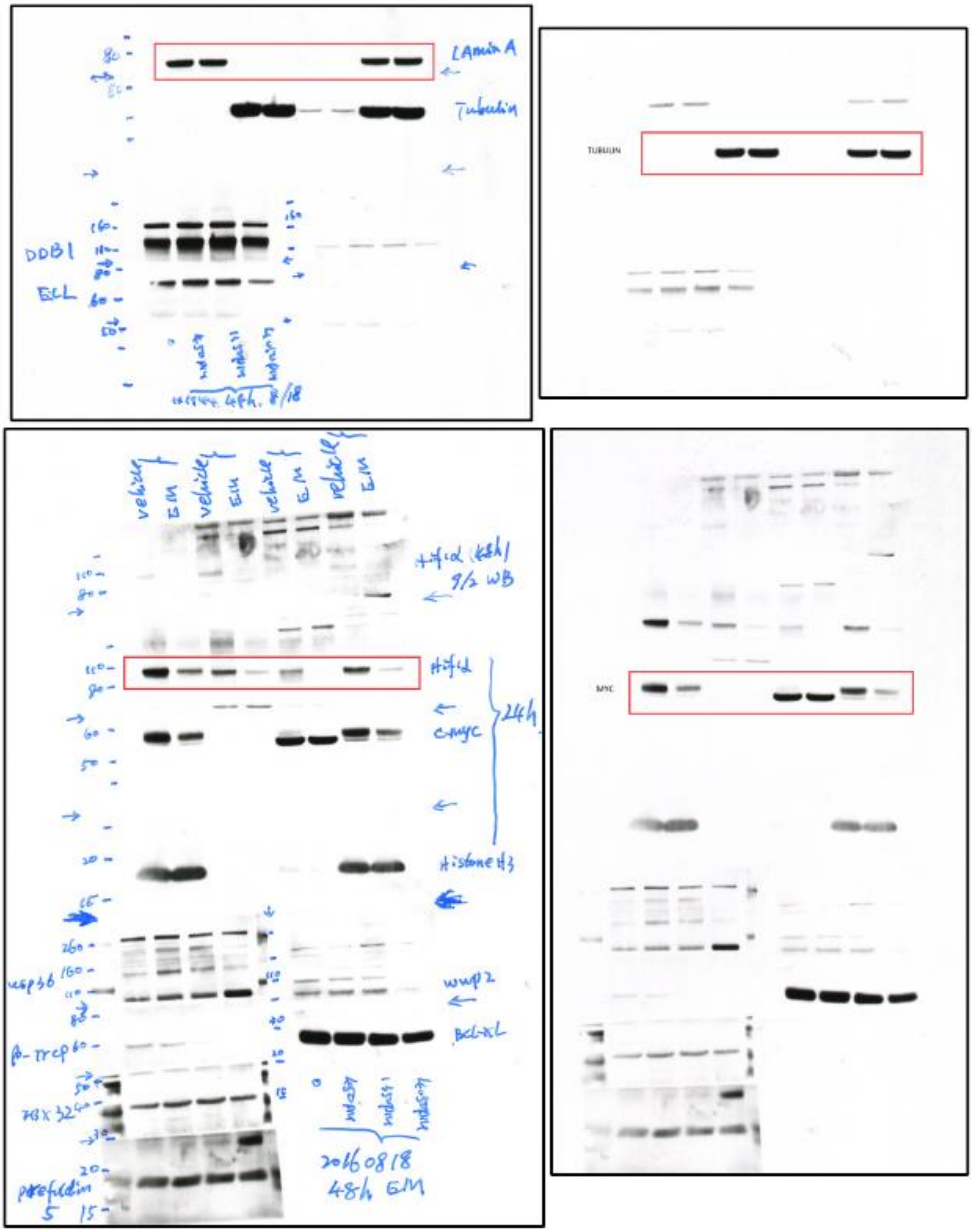
LAmin A
Tubulin
DDB1
BCL
TUBULIN
vehicle
EM
Hif1α
c-myc
24h
Histone H3
usp36
wwp2
Bcl-xL
β-Trcp
prefoldin 5
20160818
48h EM
MYC

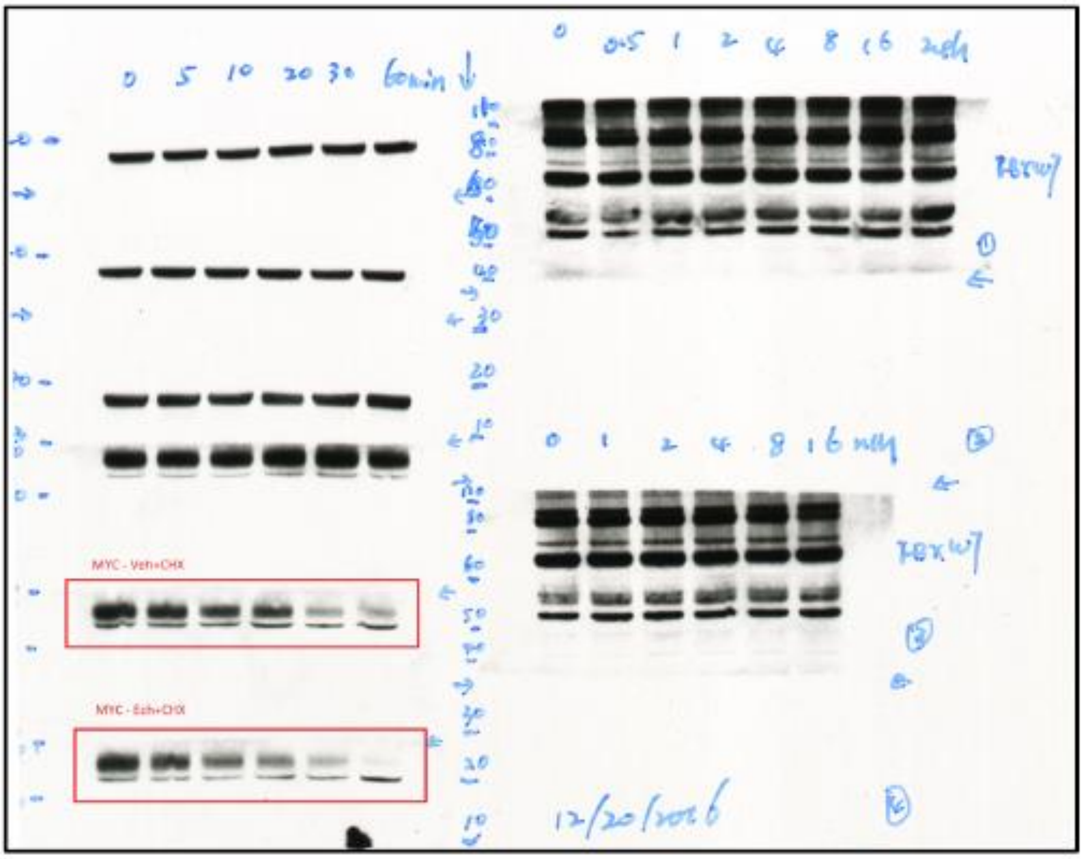
MYC - Veh+CHX
MYC - Ech+CHX

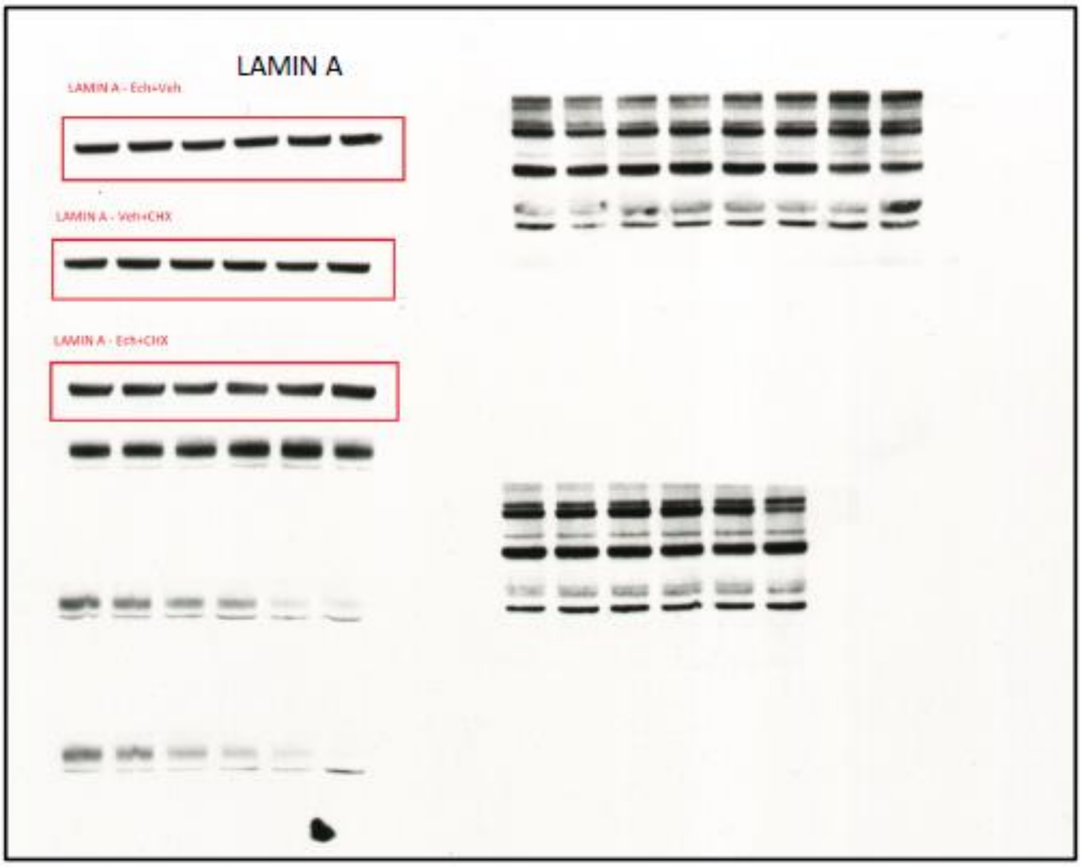
LAMIN A
LAMIN A - Ech+Veh
LAMIN A - Veh+CHX
LAMIN A - Ech+CHX

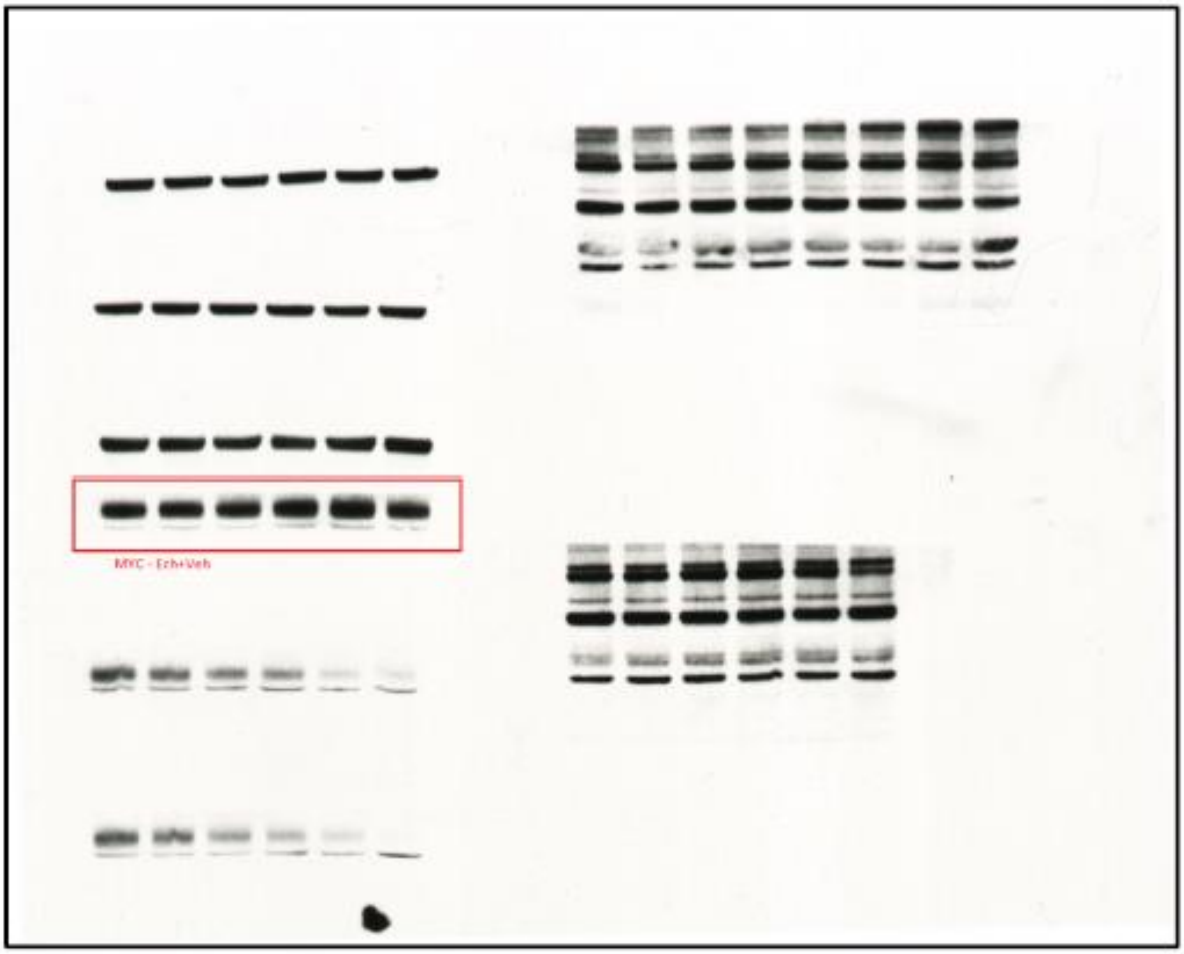
MYC - Ech+Veh

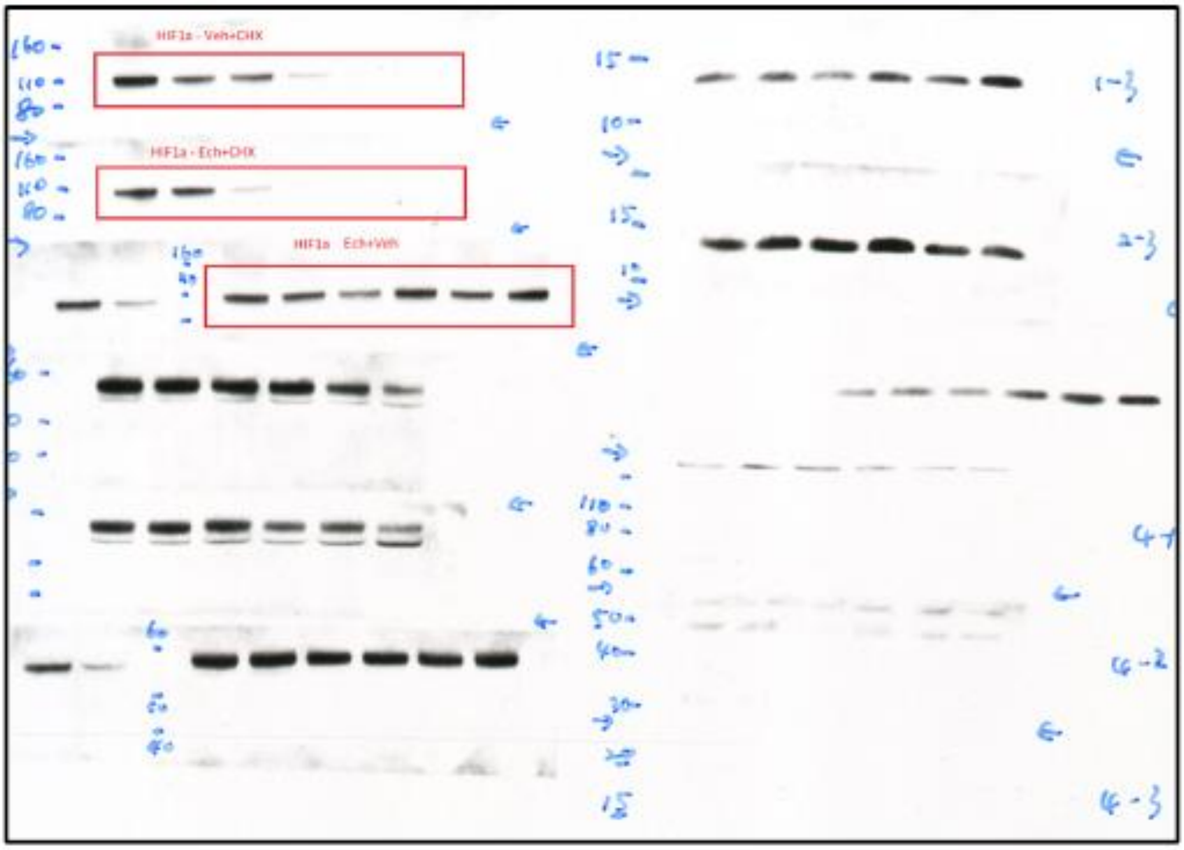
HIF1a - Veh+CHX
HIF1a - Ech+CHX
HIF1a Ech+Veh

.

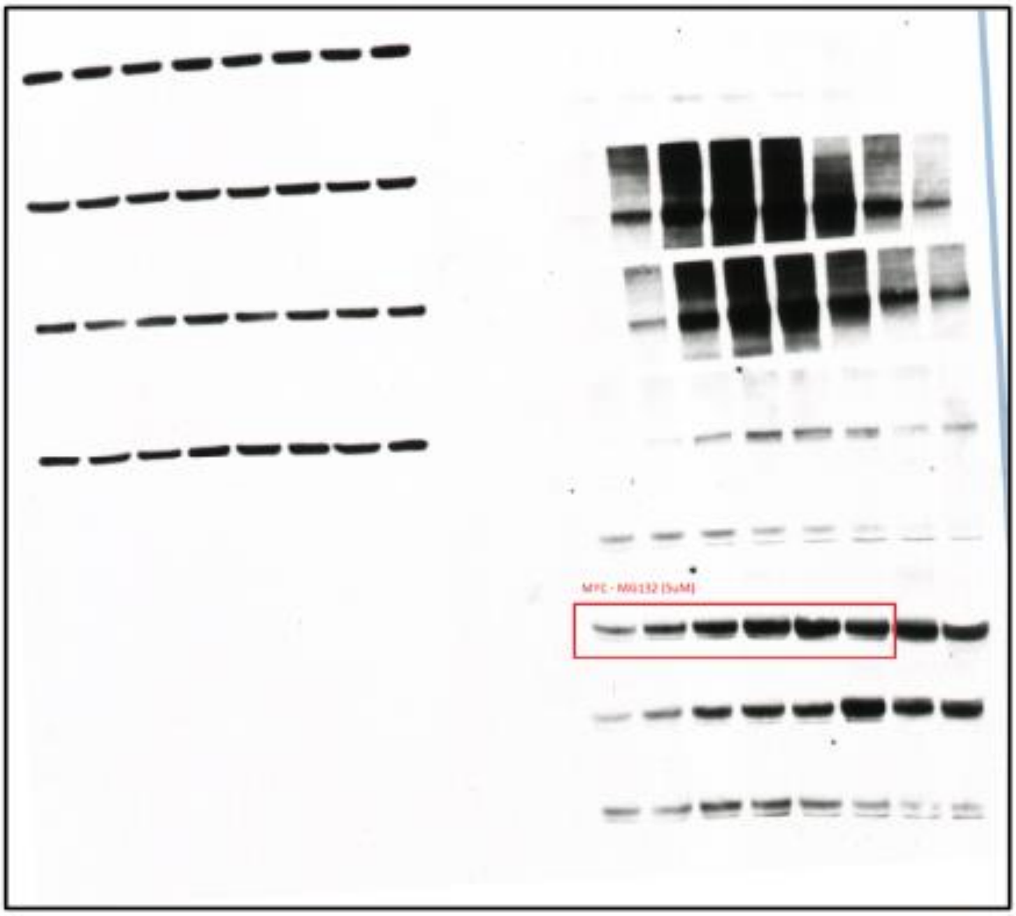
MYC - MG132 (5uM)

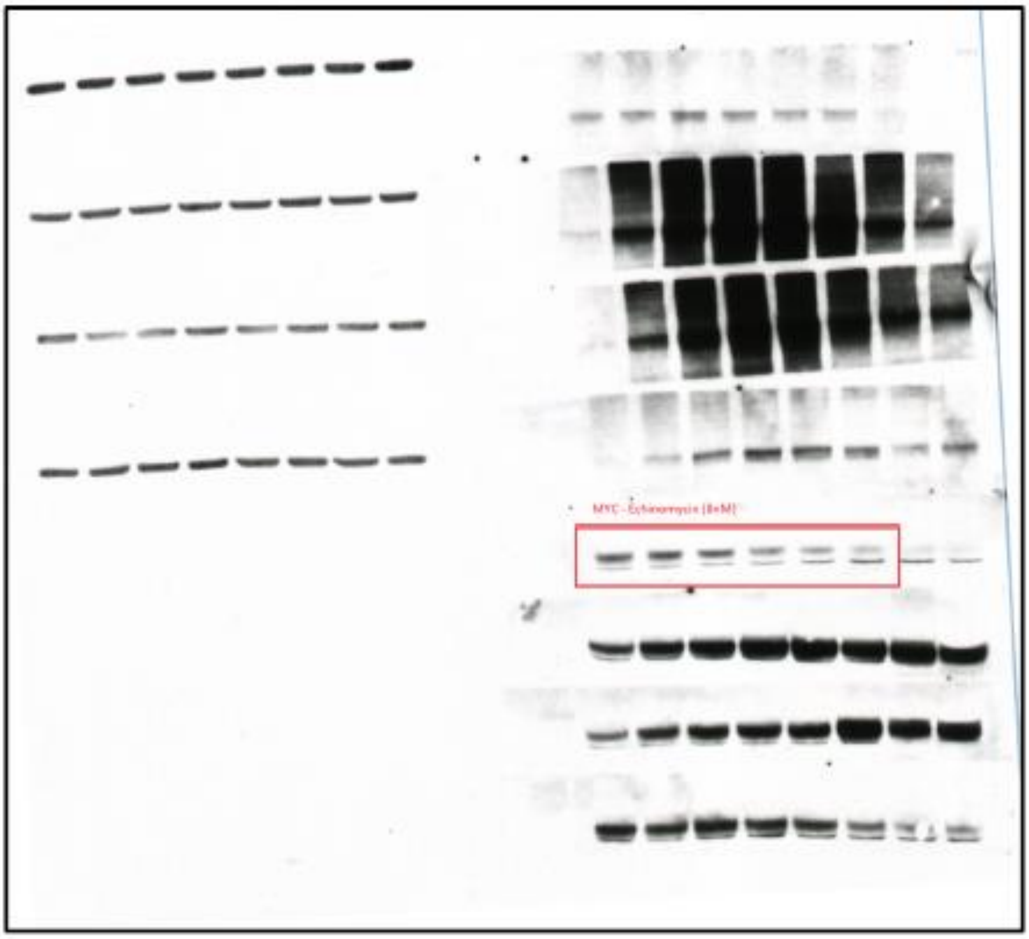
MYC - Echinomycin (8nM)

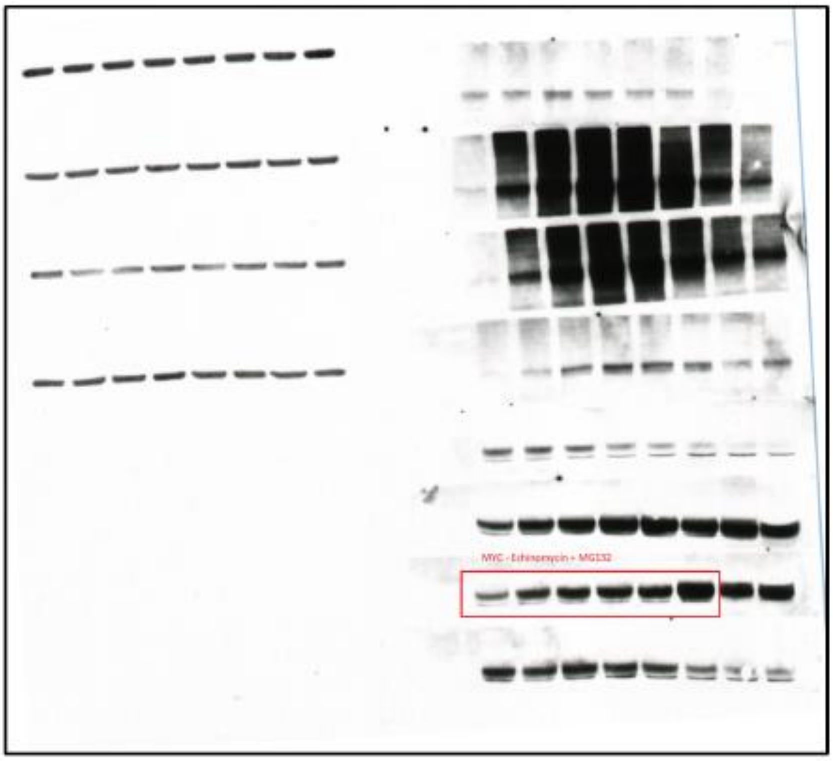
MYC - Echinomycin + MG132

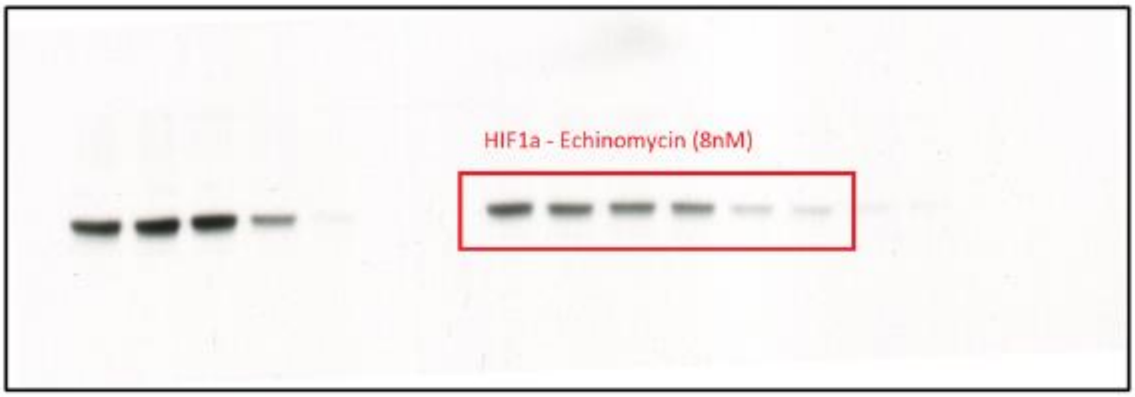
HIF1a - Echinomycin (8nM)

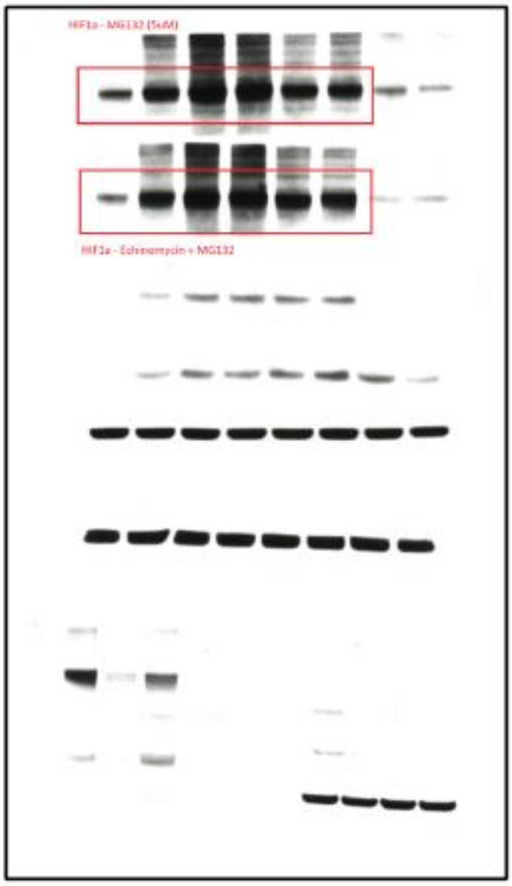

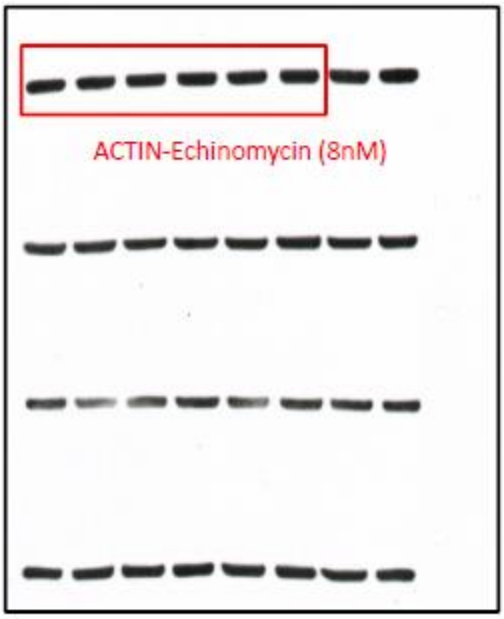
ACTIN-Echinomycin (8nM)

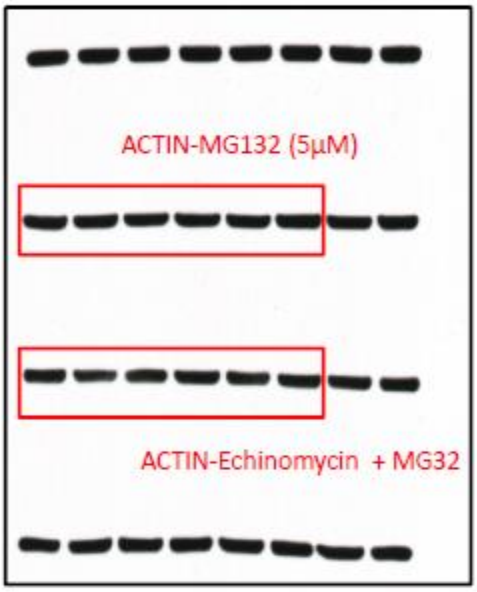
ACTIN-MG132 (5µM)
ACTIN-Echinomycin + MG32

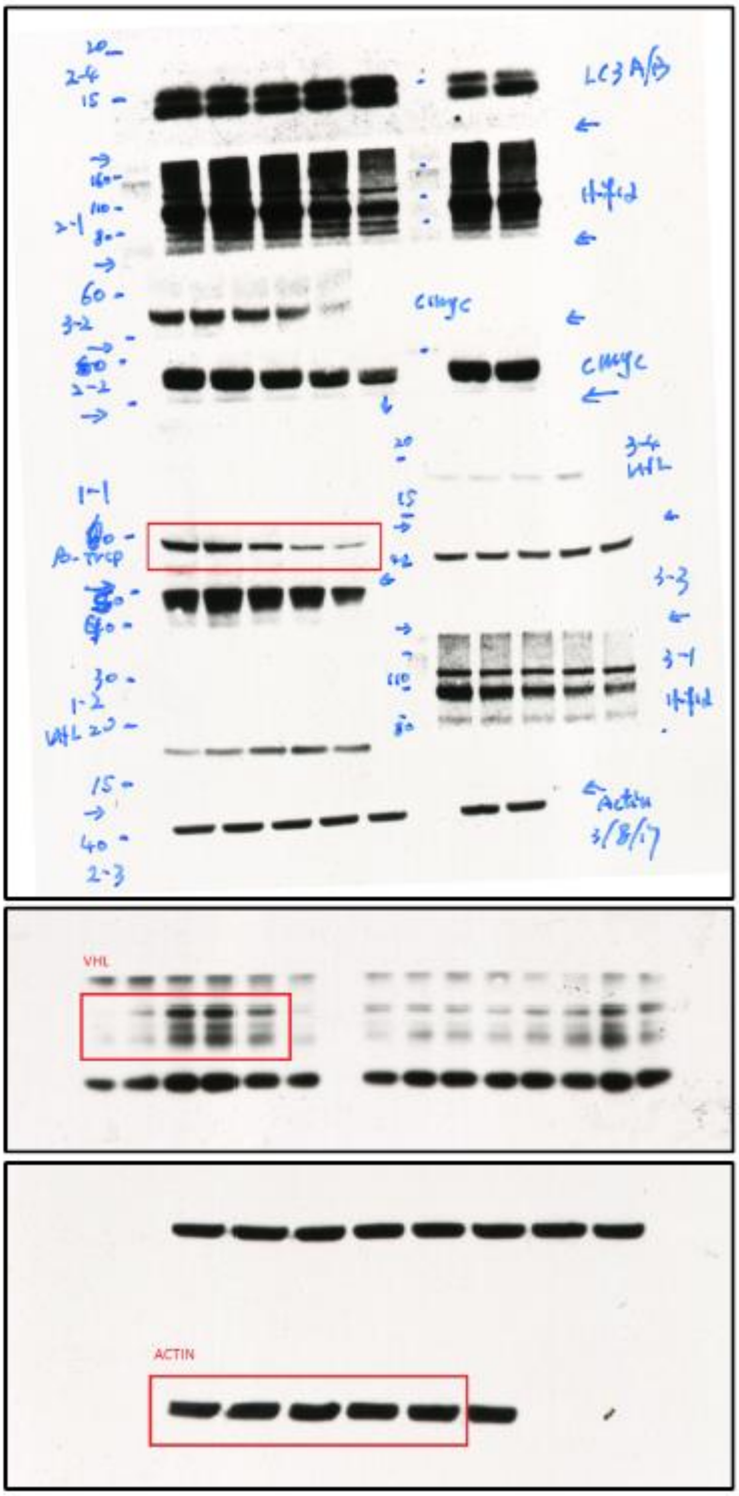
LC3 A/B
cmyc
VHL
ACTIN

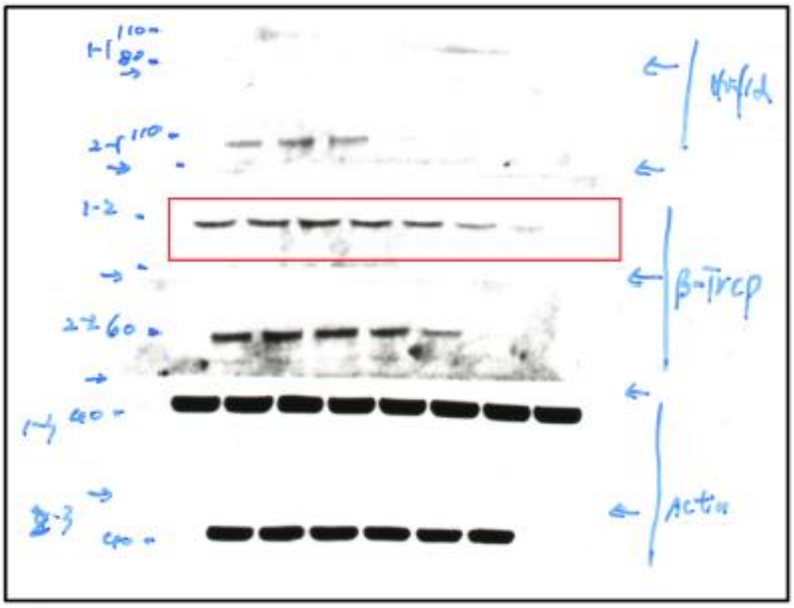

β-Trcp
Actin

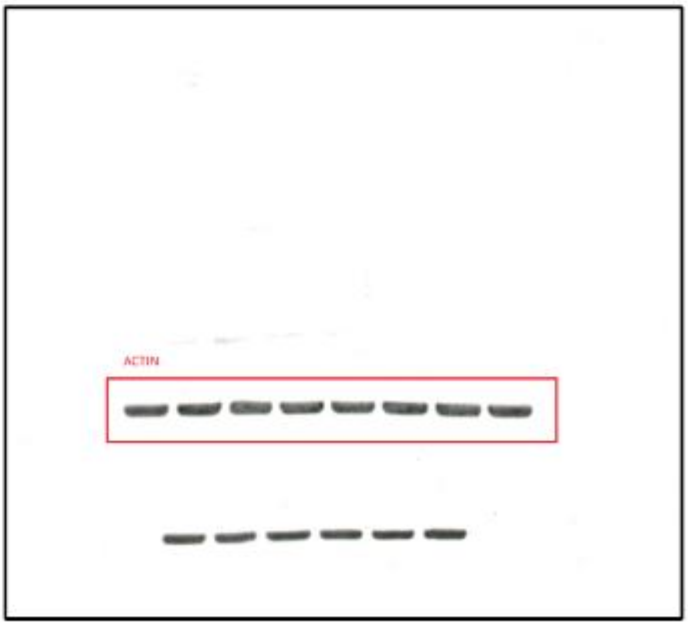

ACTIN

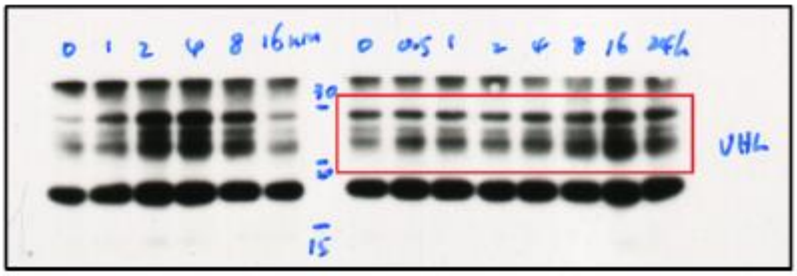

VHL
15

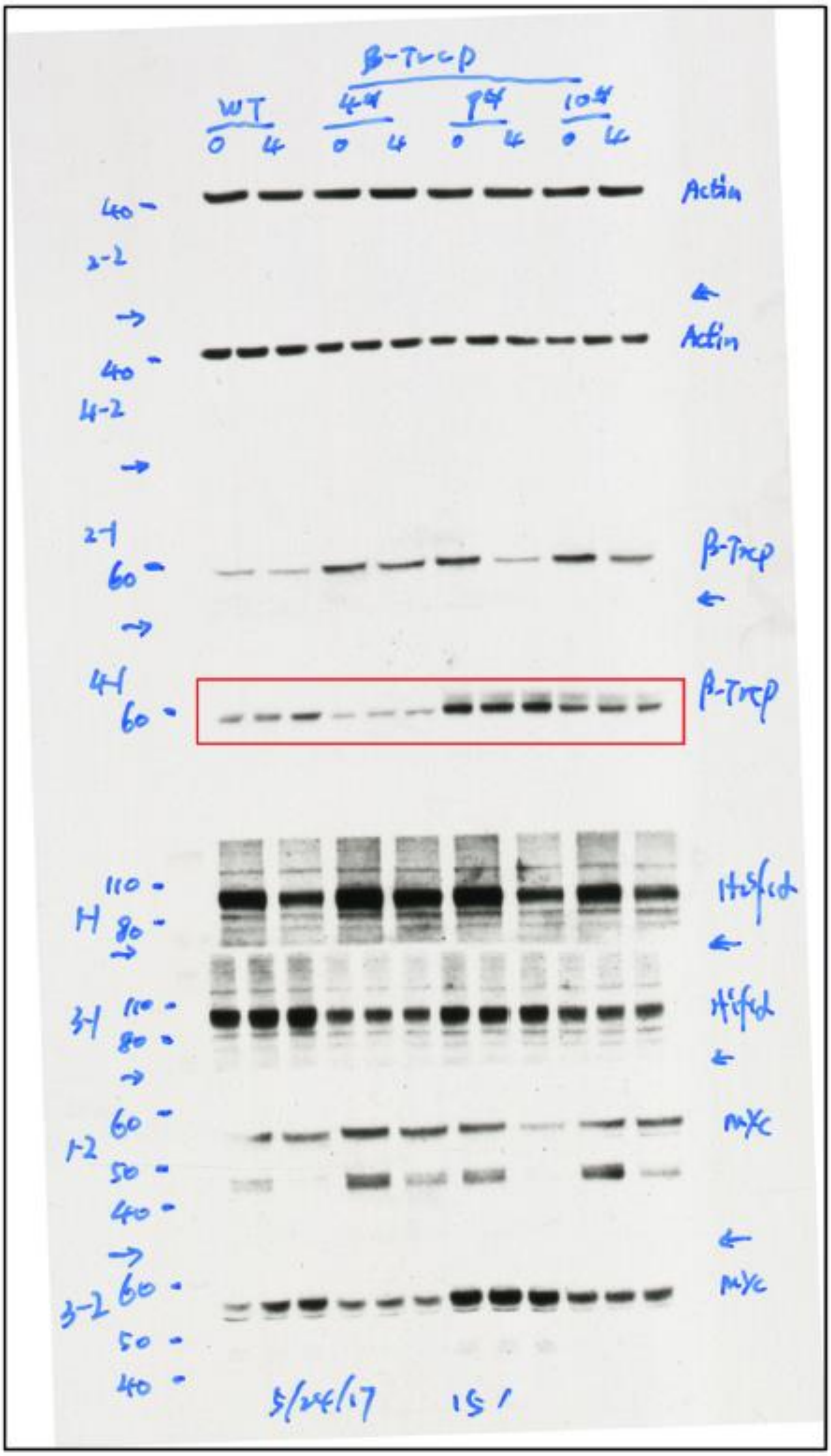
β-Trcp
WT
Actin
Actin
β-Trcp
β-Trcp
myc
myc

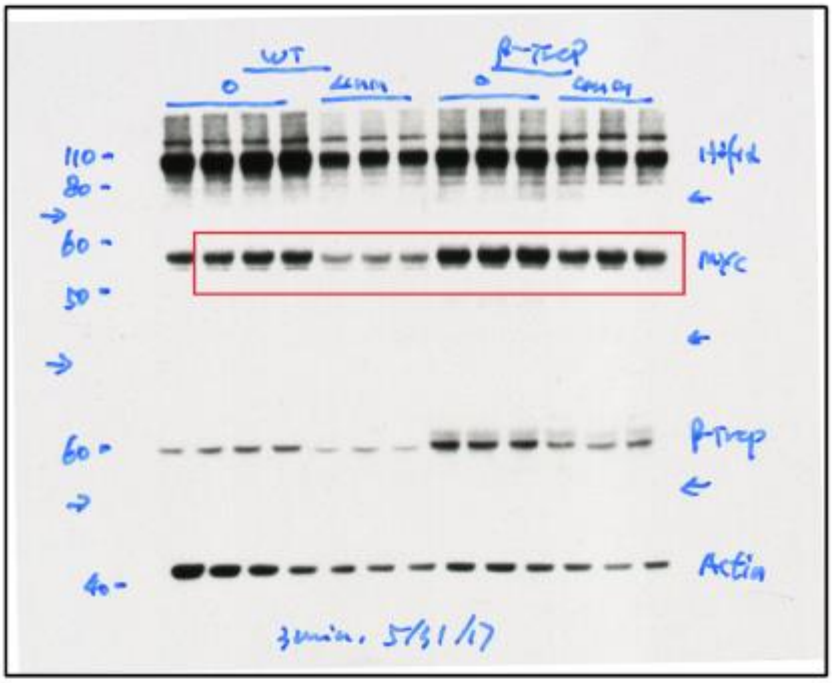
WT
β-Trcp
110
80
60
50
Myc
β-Trcp
Actin
40

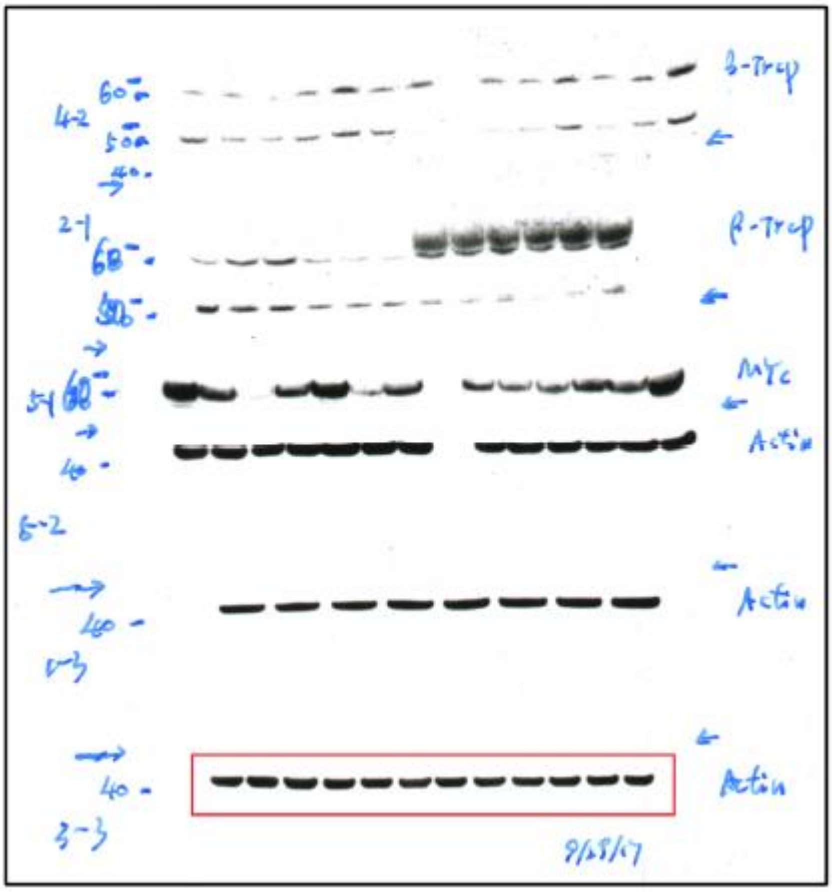
β-Trcp
β-Trcp
Myc
Actin
Actin
Actin
40

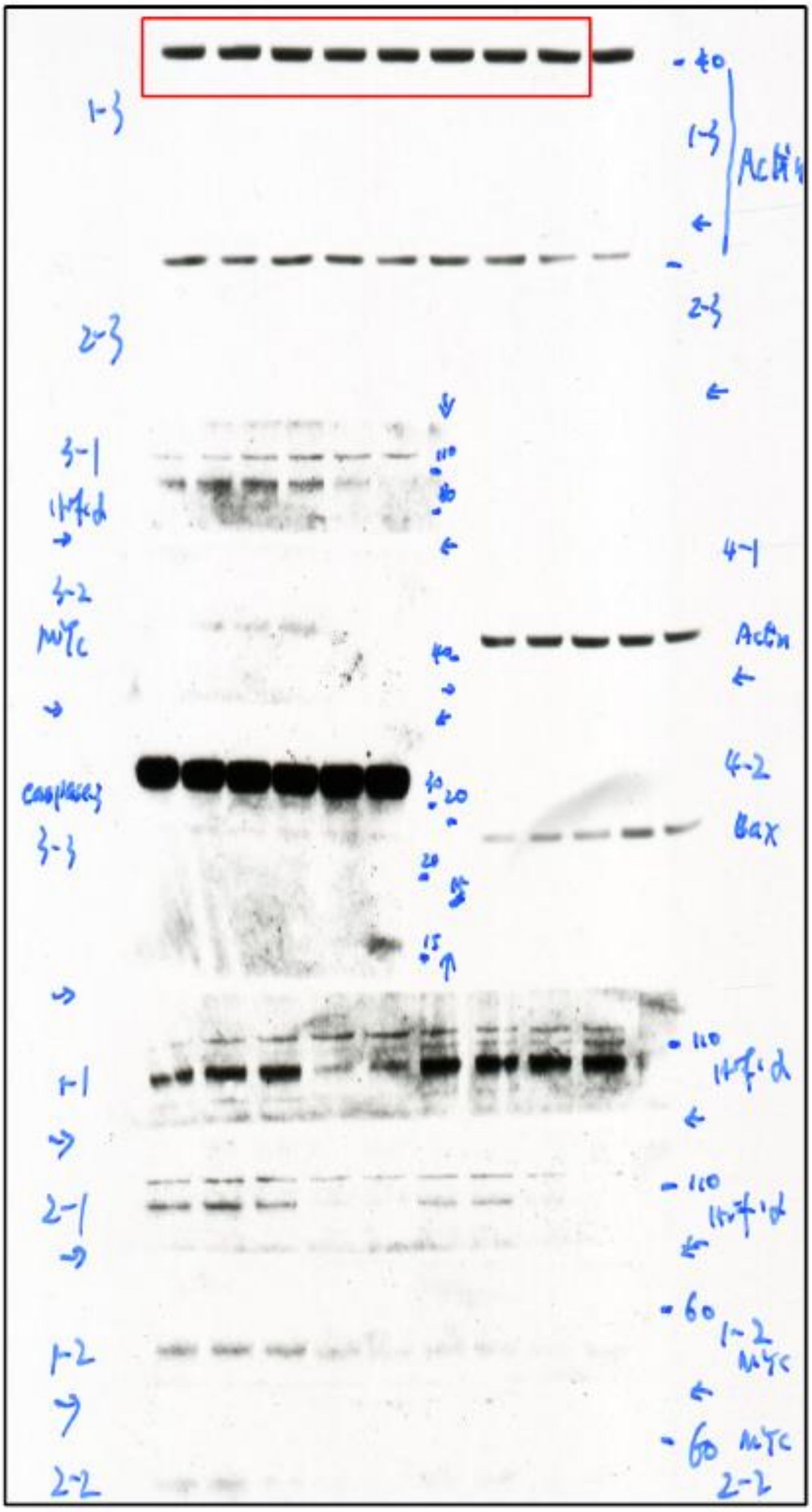
1-3
Actin
40
2-3
3-1
3-2
4-1
Actin
4-2
Bax
3-3
1-1
110
2-1
110
60
1-2
60
2-2

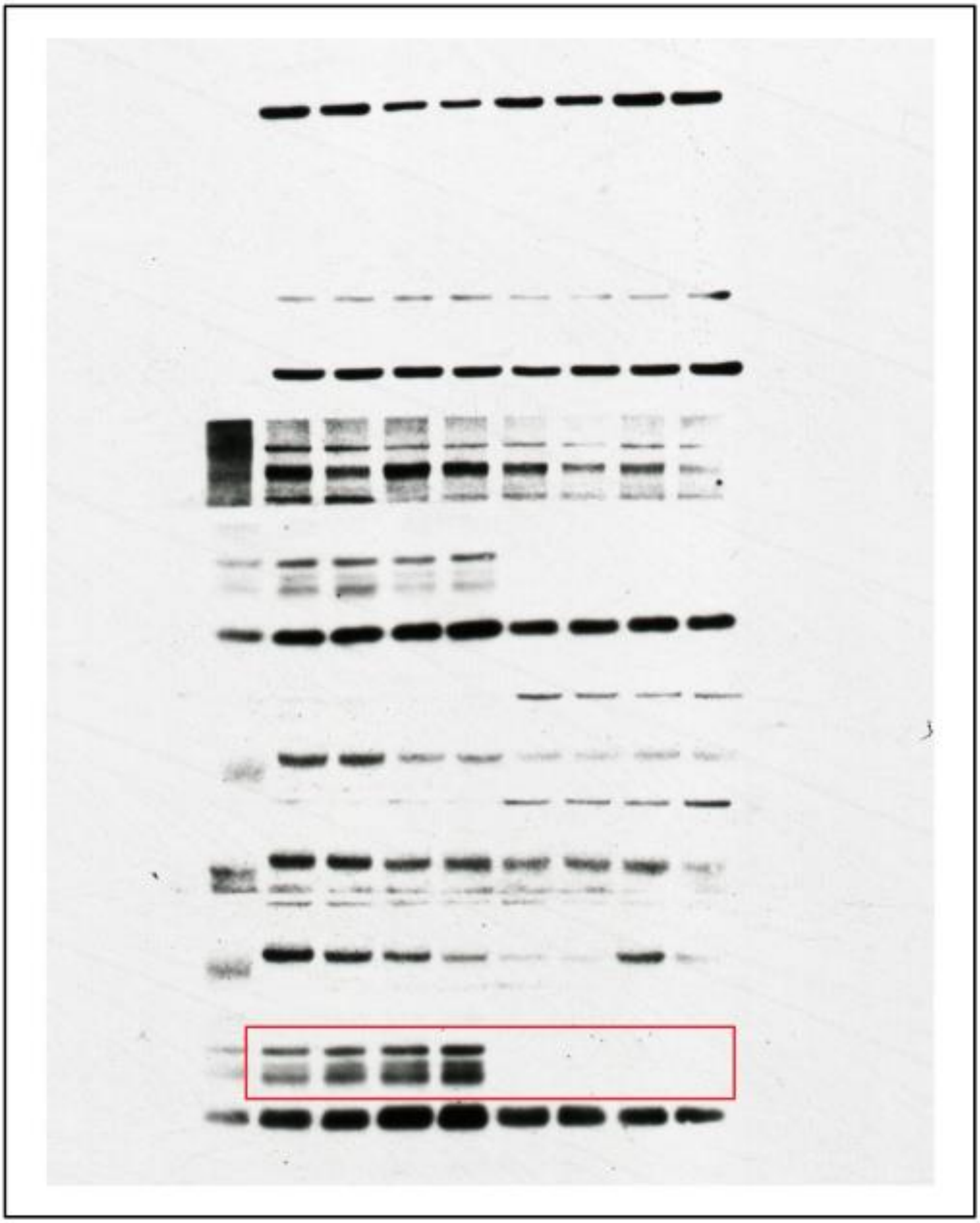

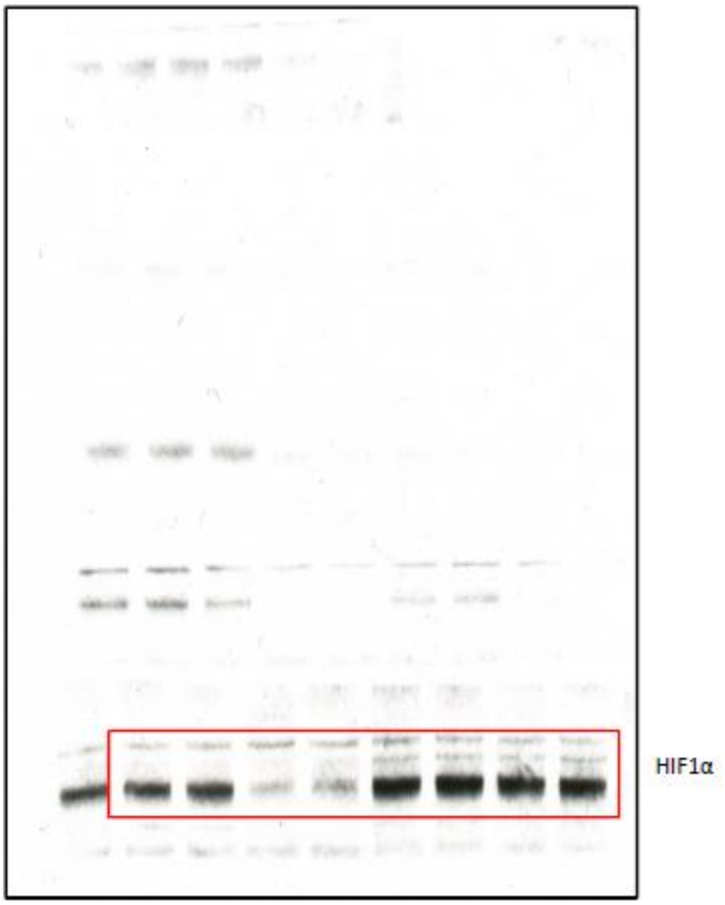
HIF1α

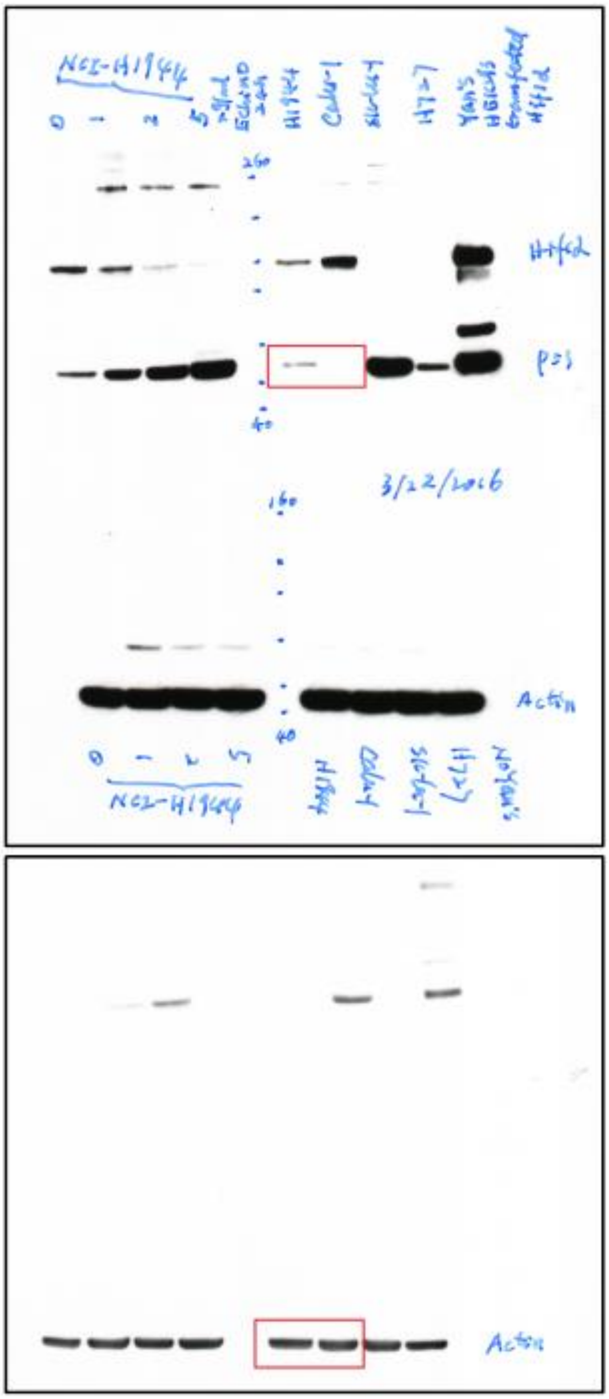
NCI-H1944
0 1 2 5
p53
40
160
3/22/2016
Actin
Actin

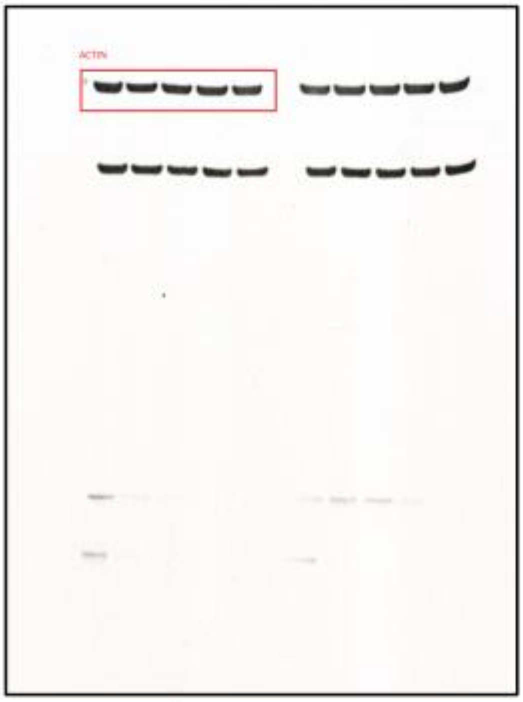
ACTIN

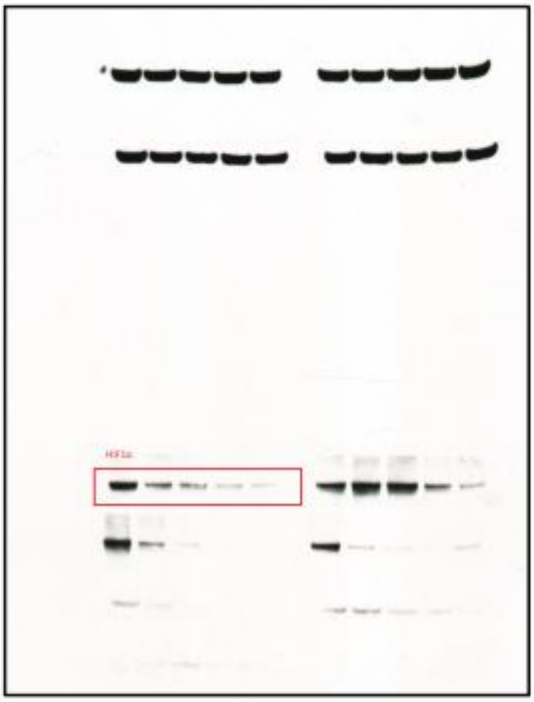
HIF1α

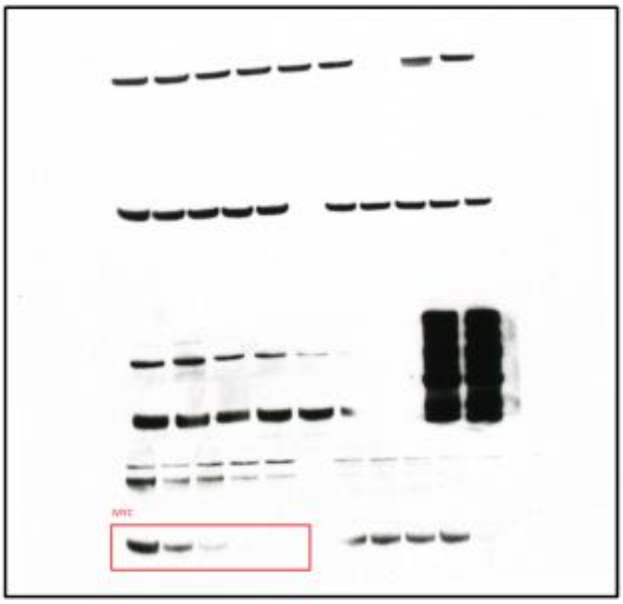

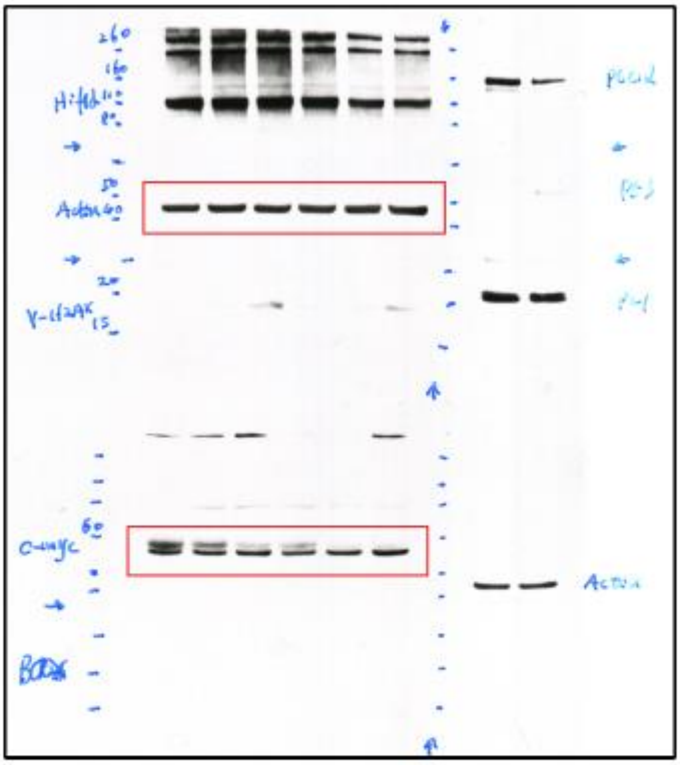
260
160
Actin
c-myc
Actin

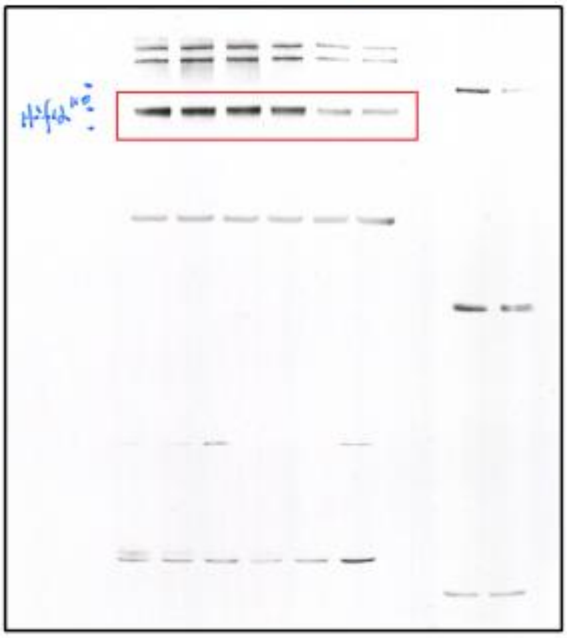

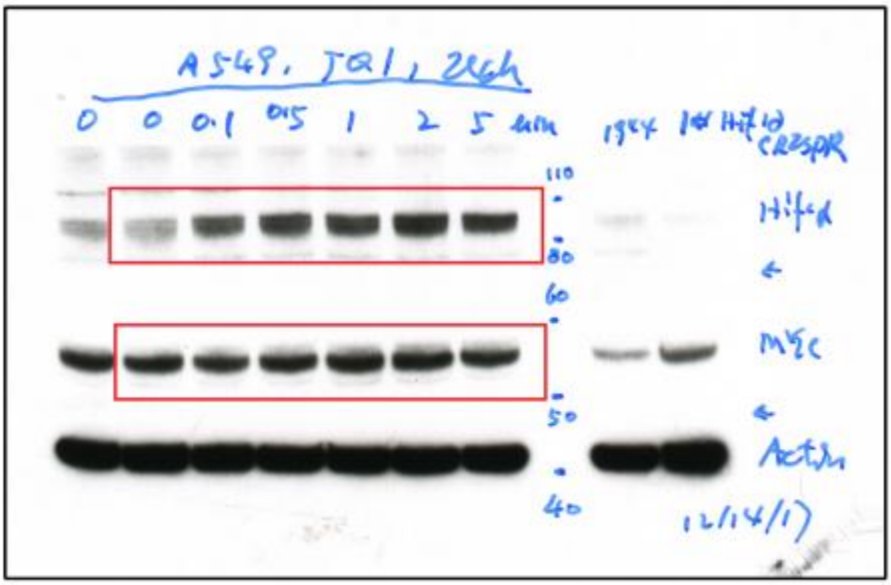
110
80
60
50
40
Myc
Actin

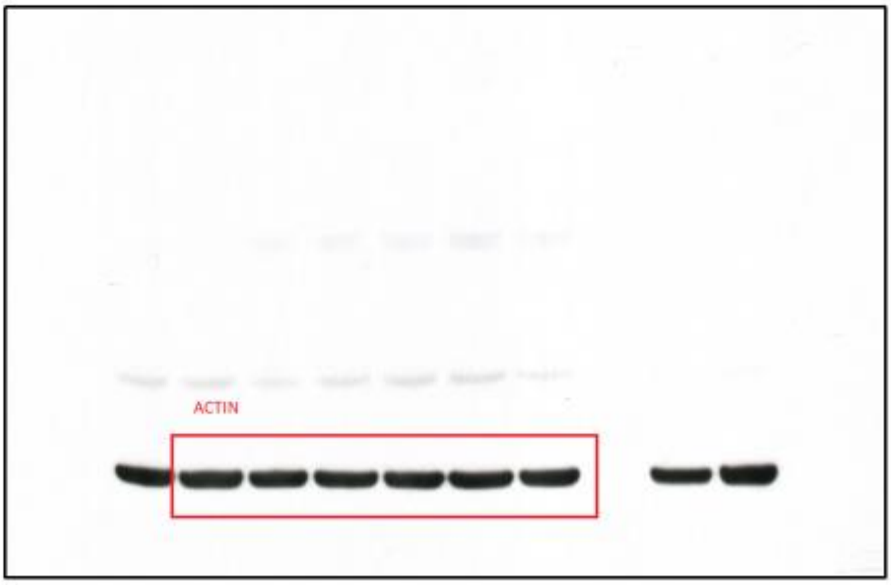
ACTIN

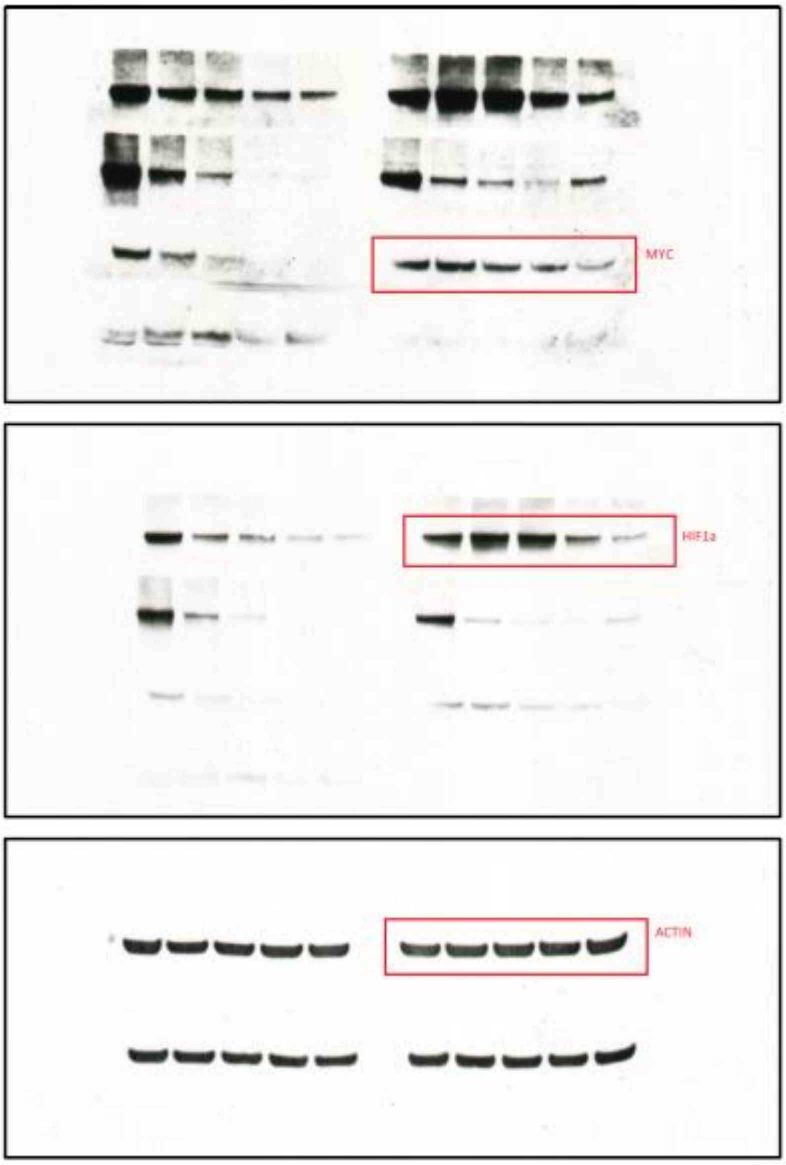
MYC
HIF1a
ACTIN

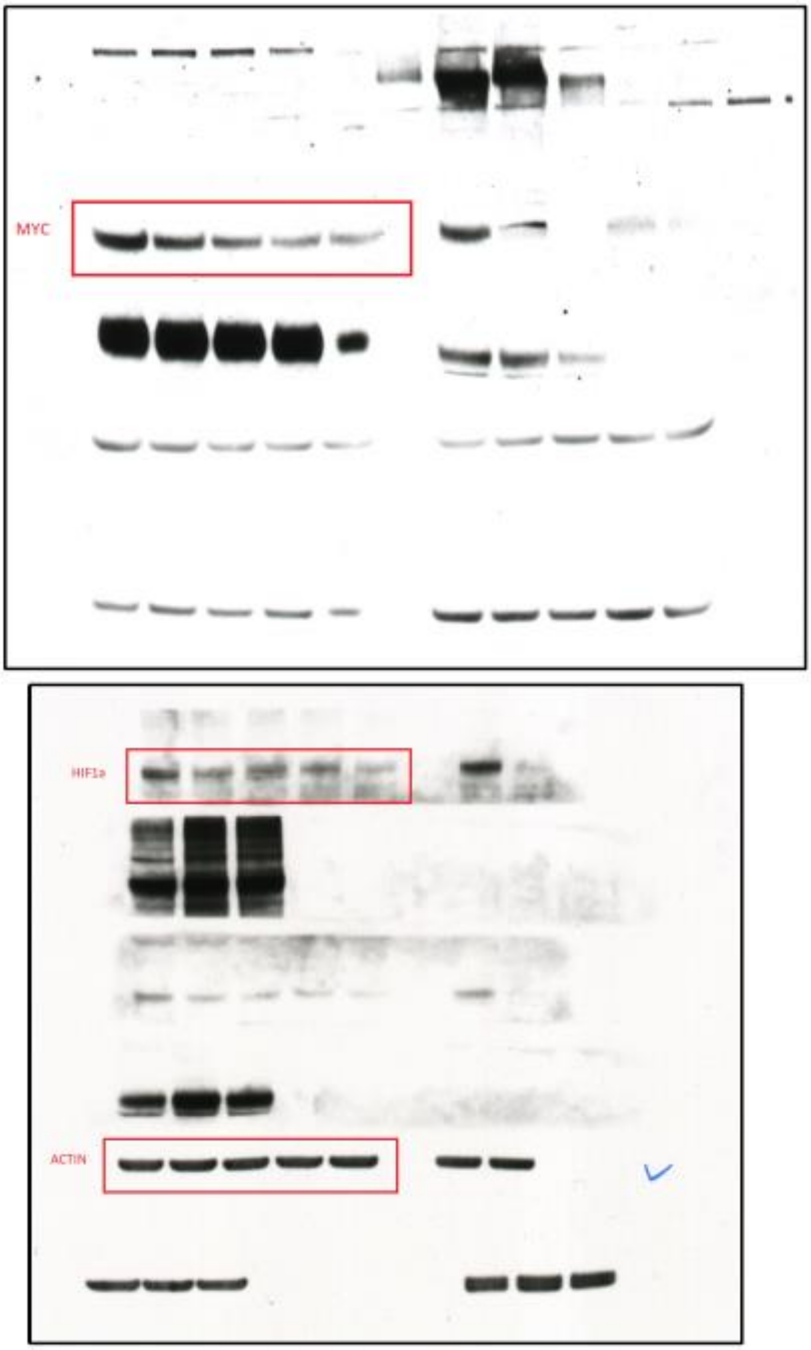

Fig S4A

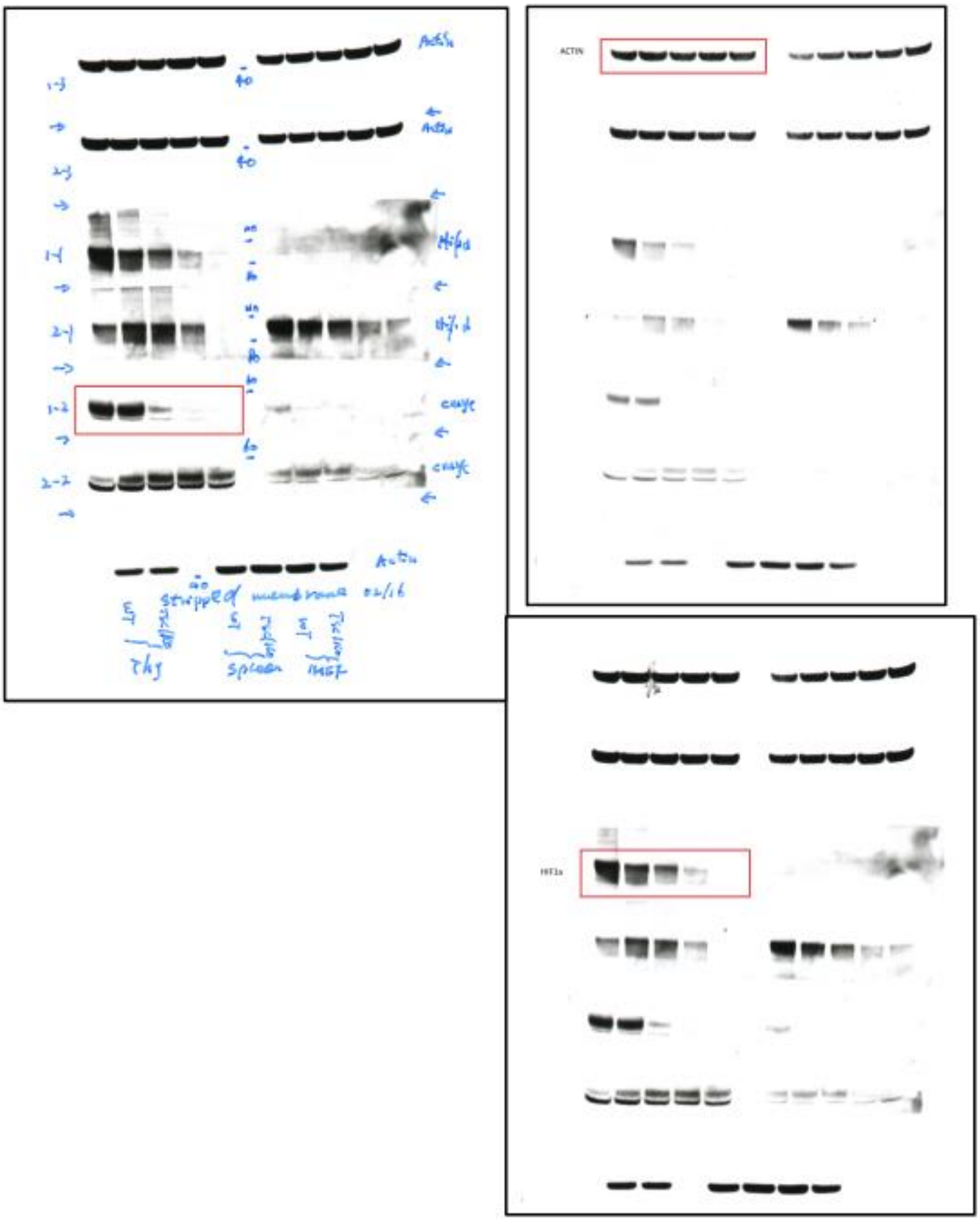

Fig S4B

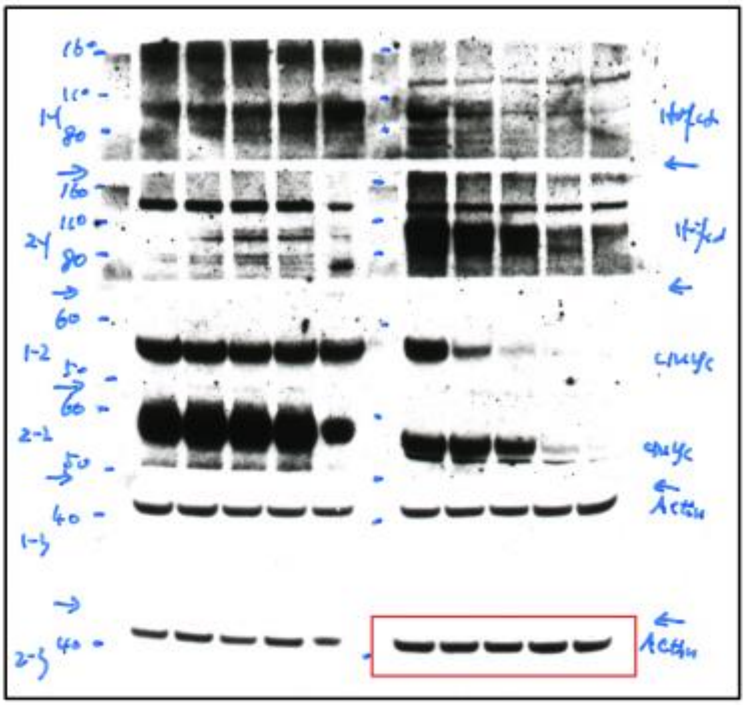

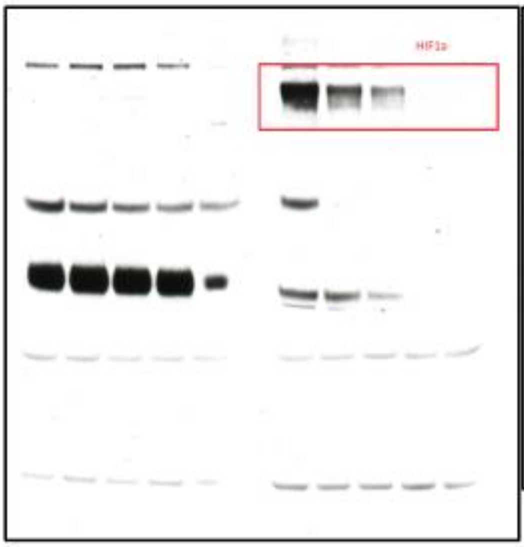

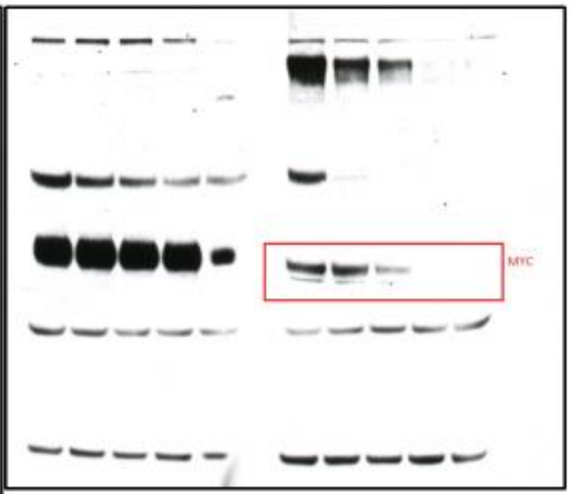
MYC

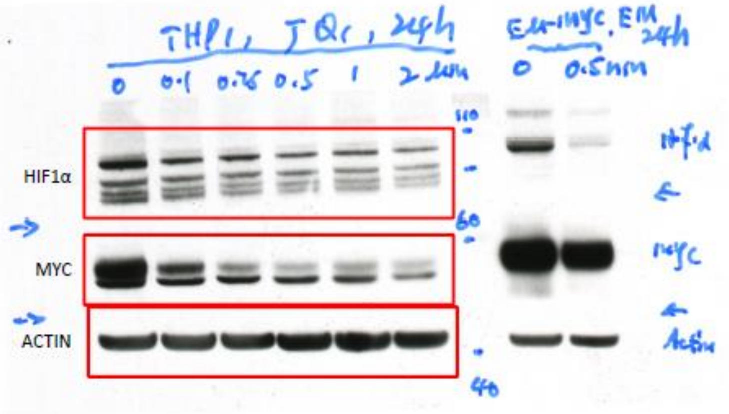
HIF1α
MYC
ACTIN

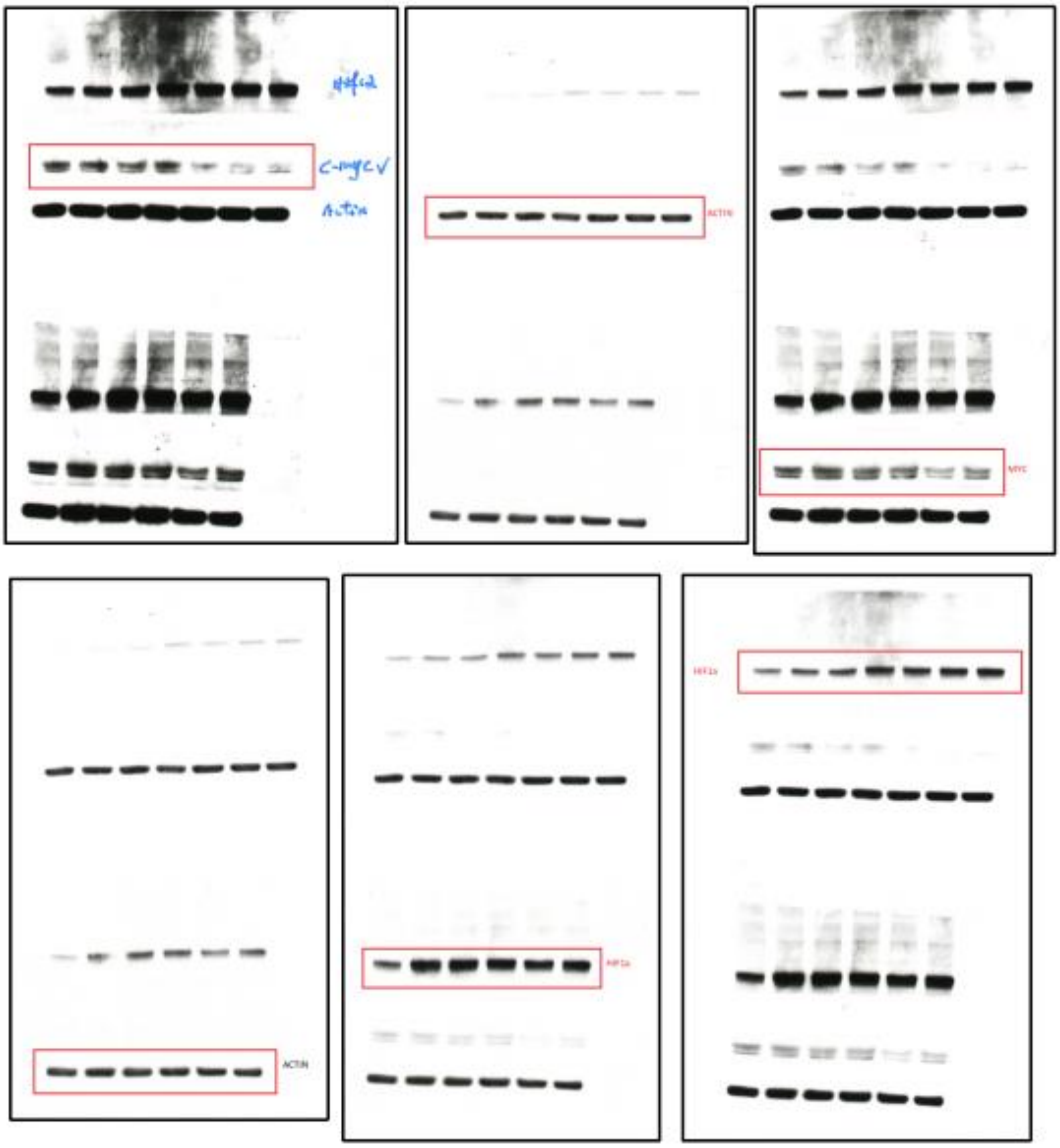
Actin
ACTIN

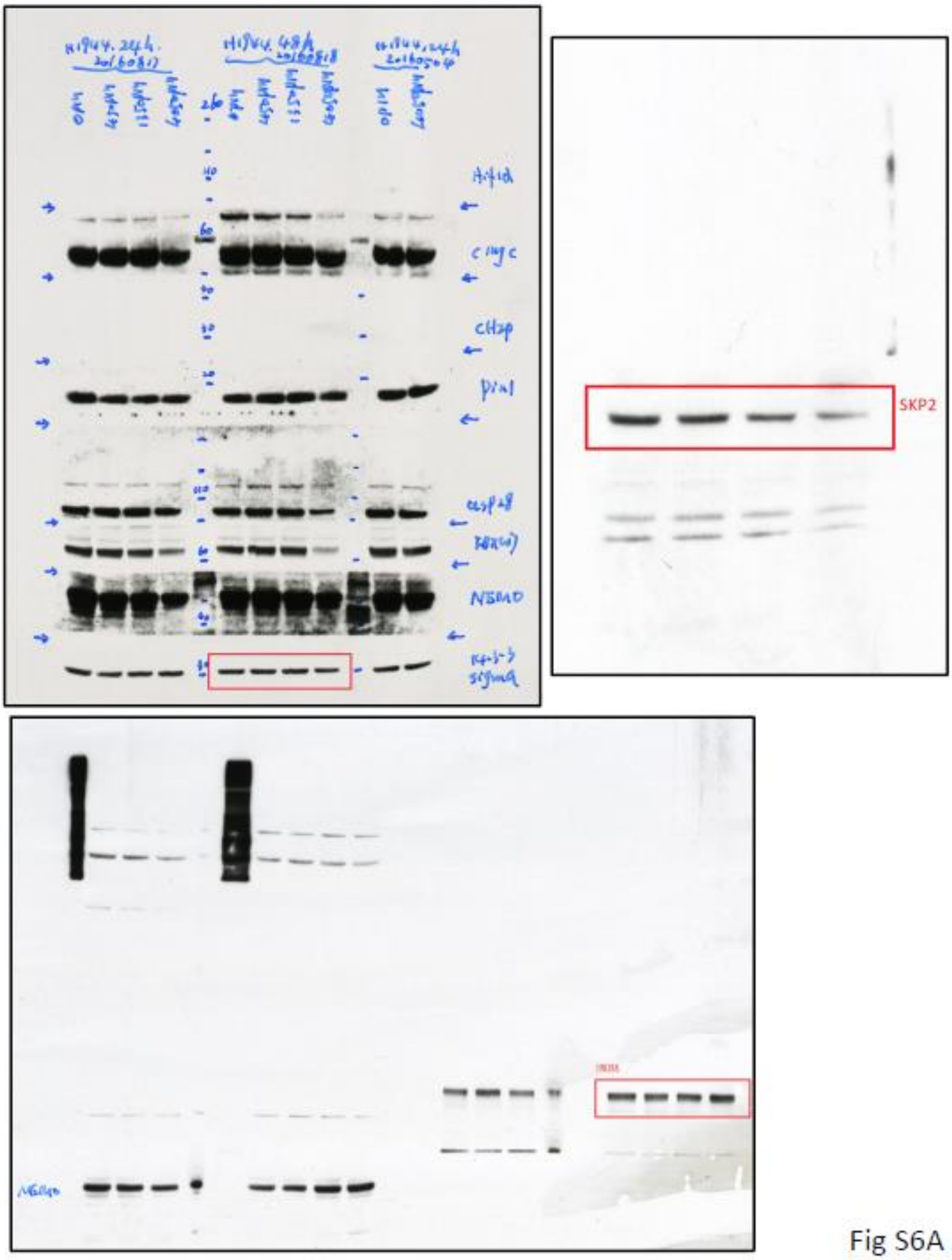

Fig S6A

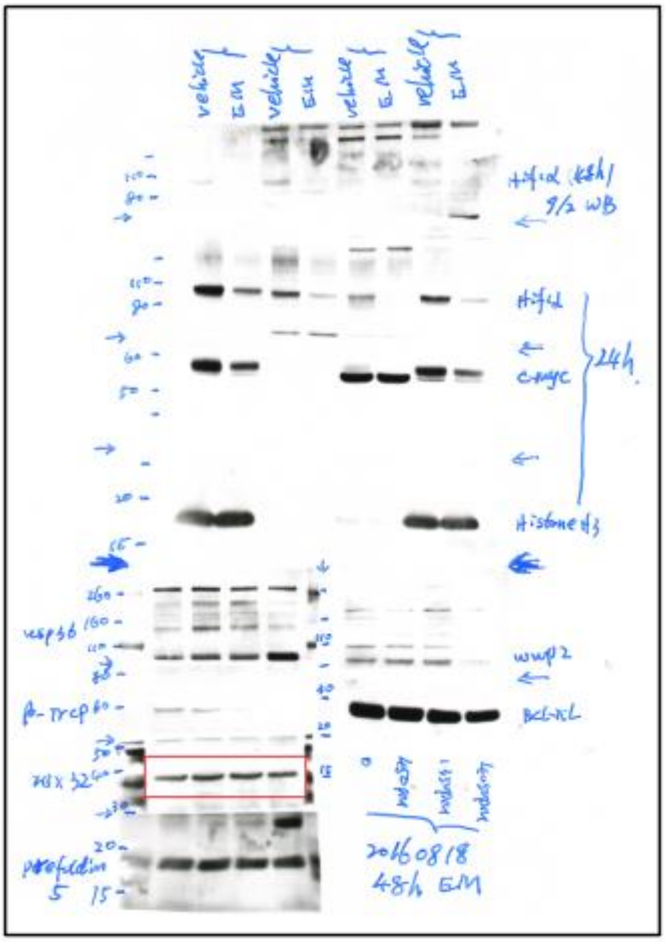

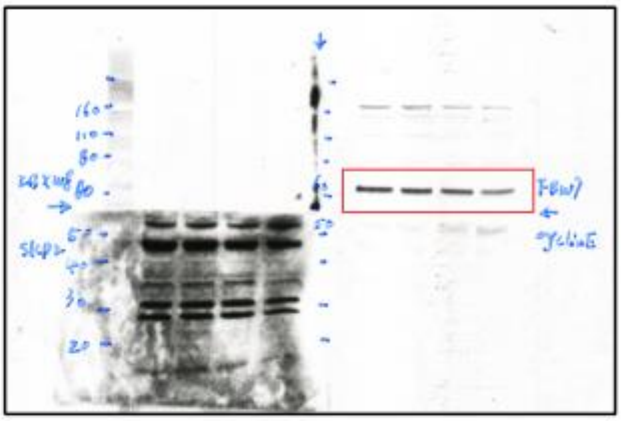

Fig S6A

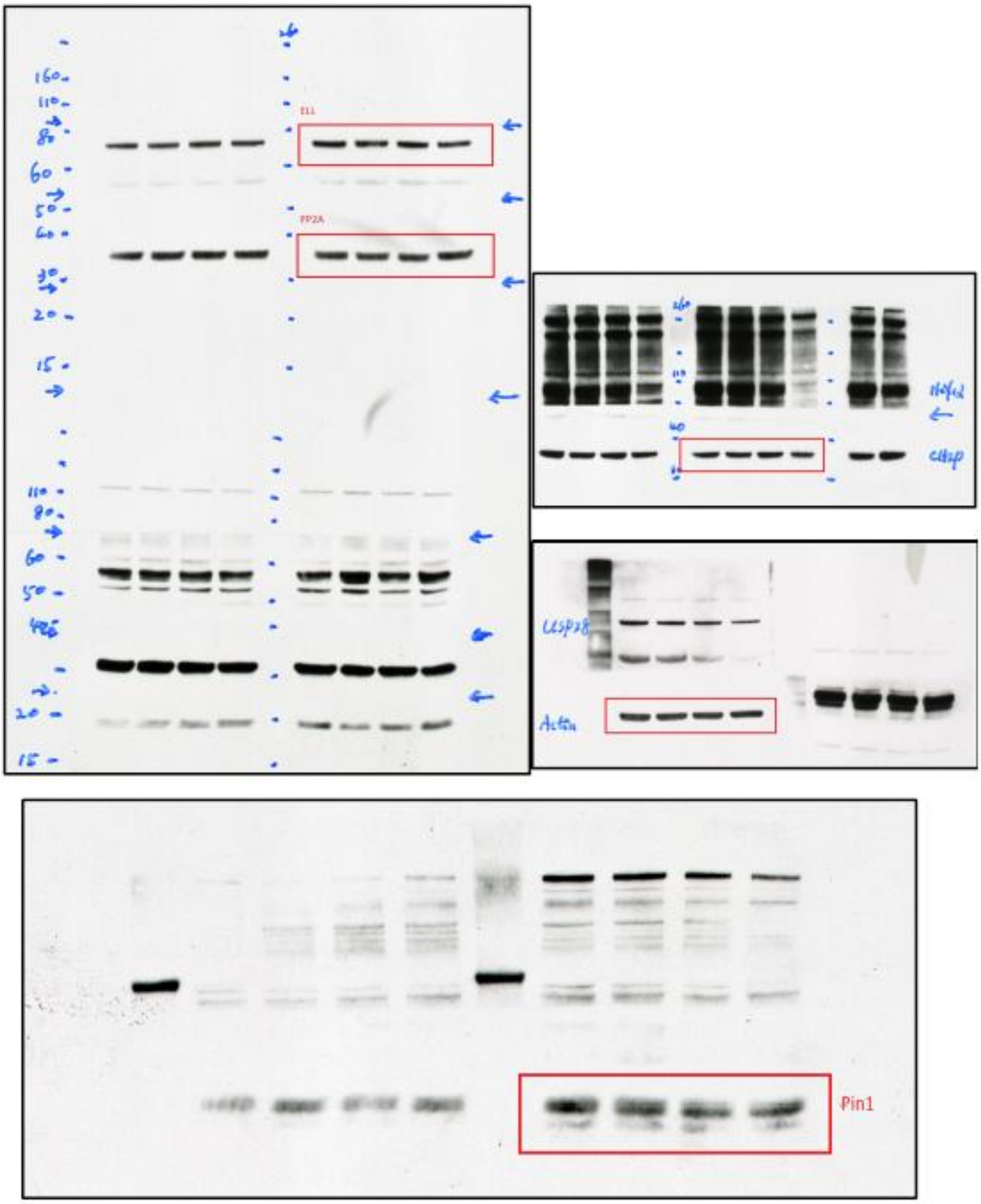

Fig S6A

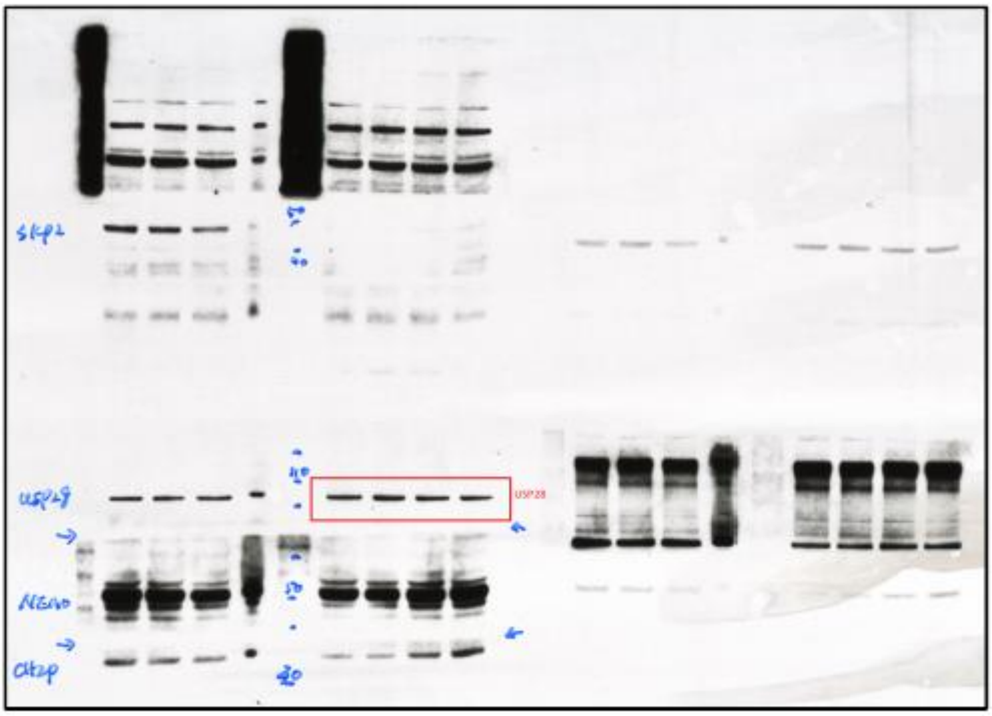
USP28
NEMO

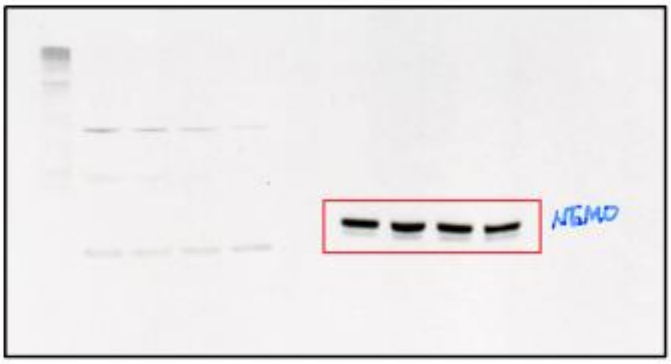
NEMO

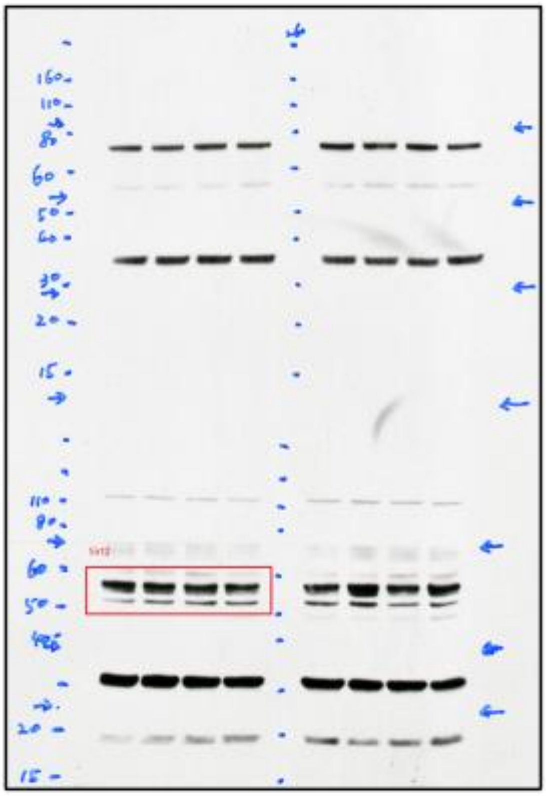
160
110
80
60
50
40
30
20
15
110
80
60
50
40
20
15
Sirt2

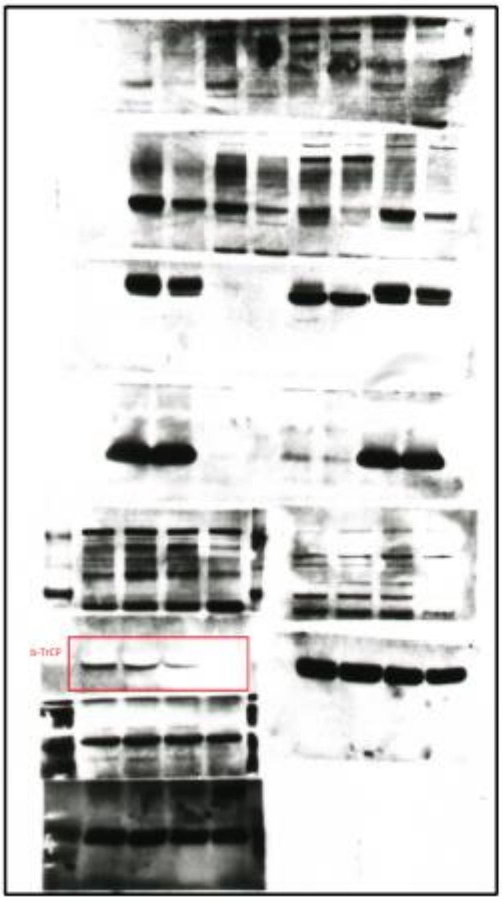
b-TrCP

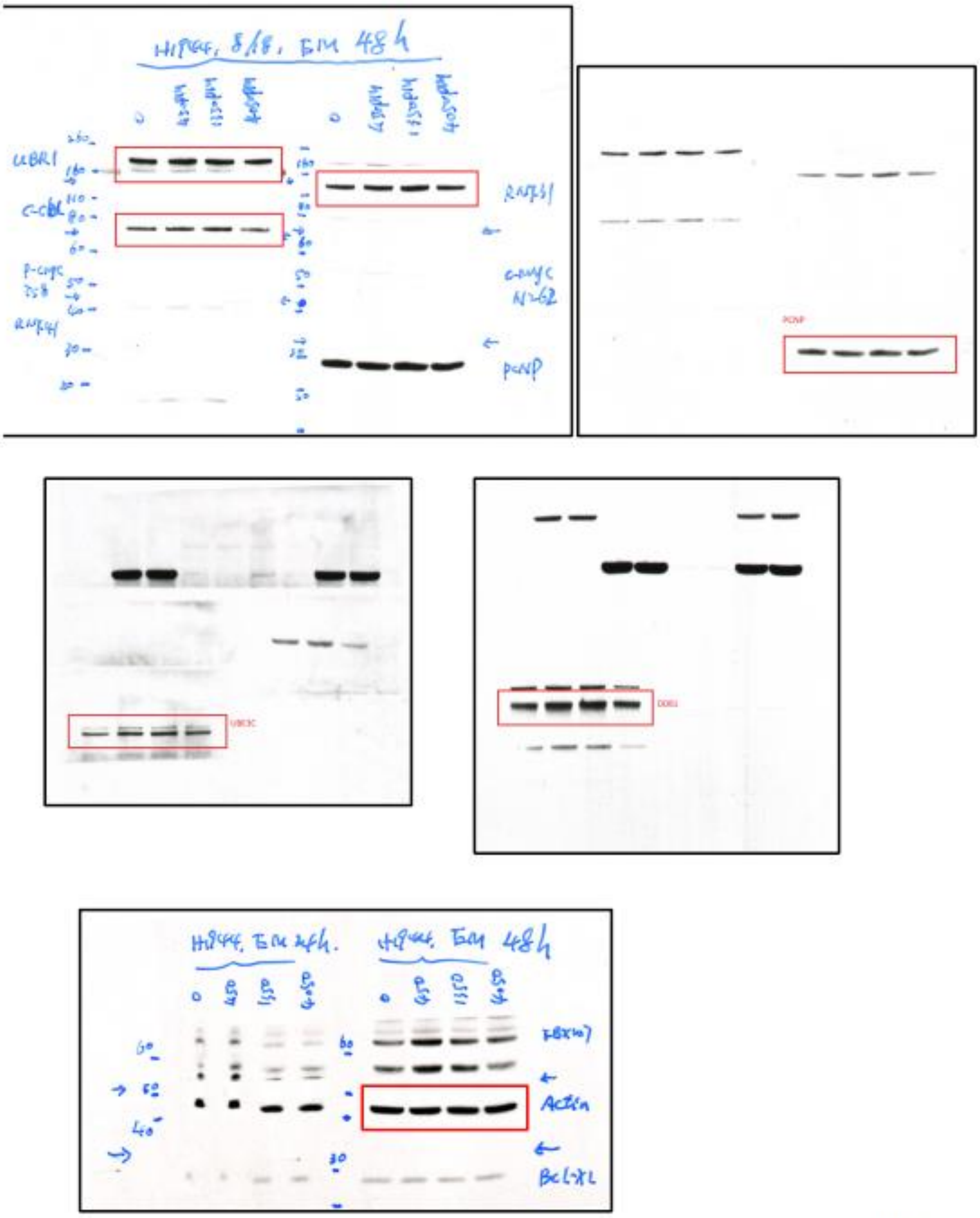

Fig S6C

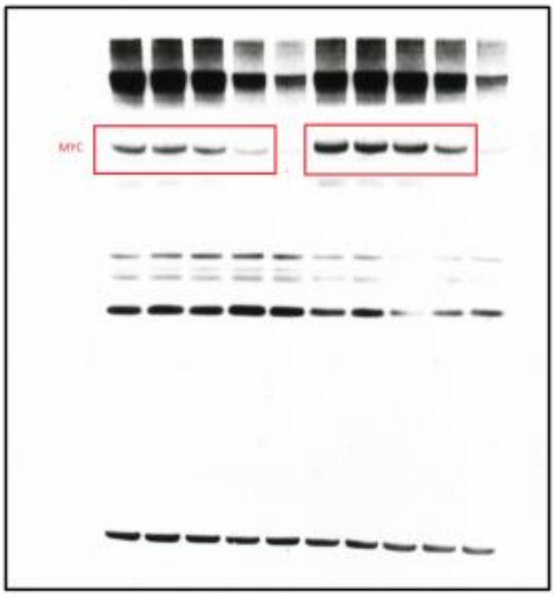
MYC

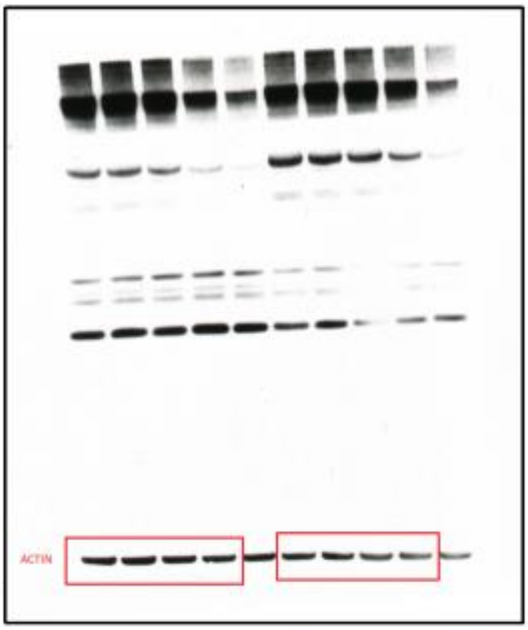
ACTIN

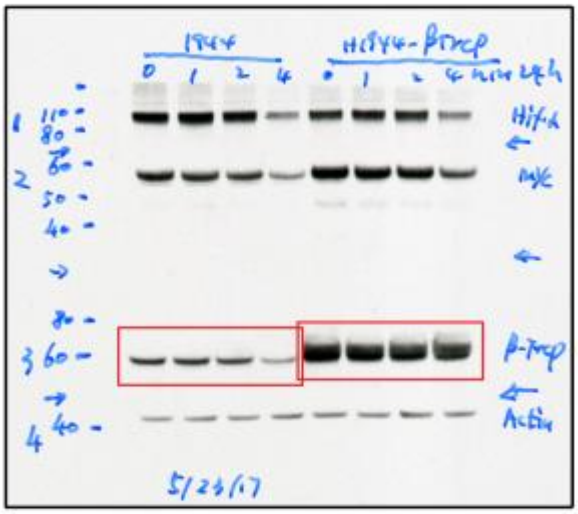
β-Trcp
Actin

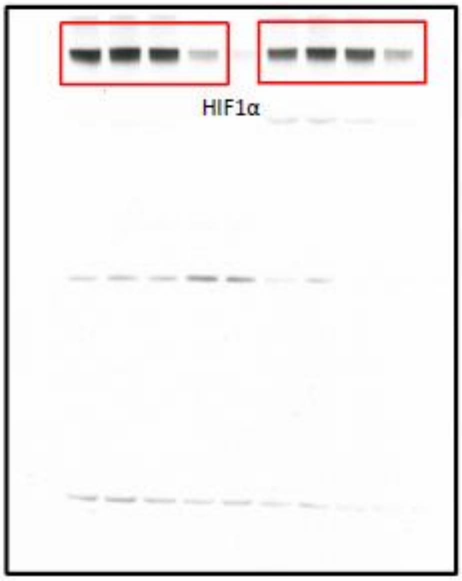
HIF1α

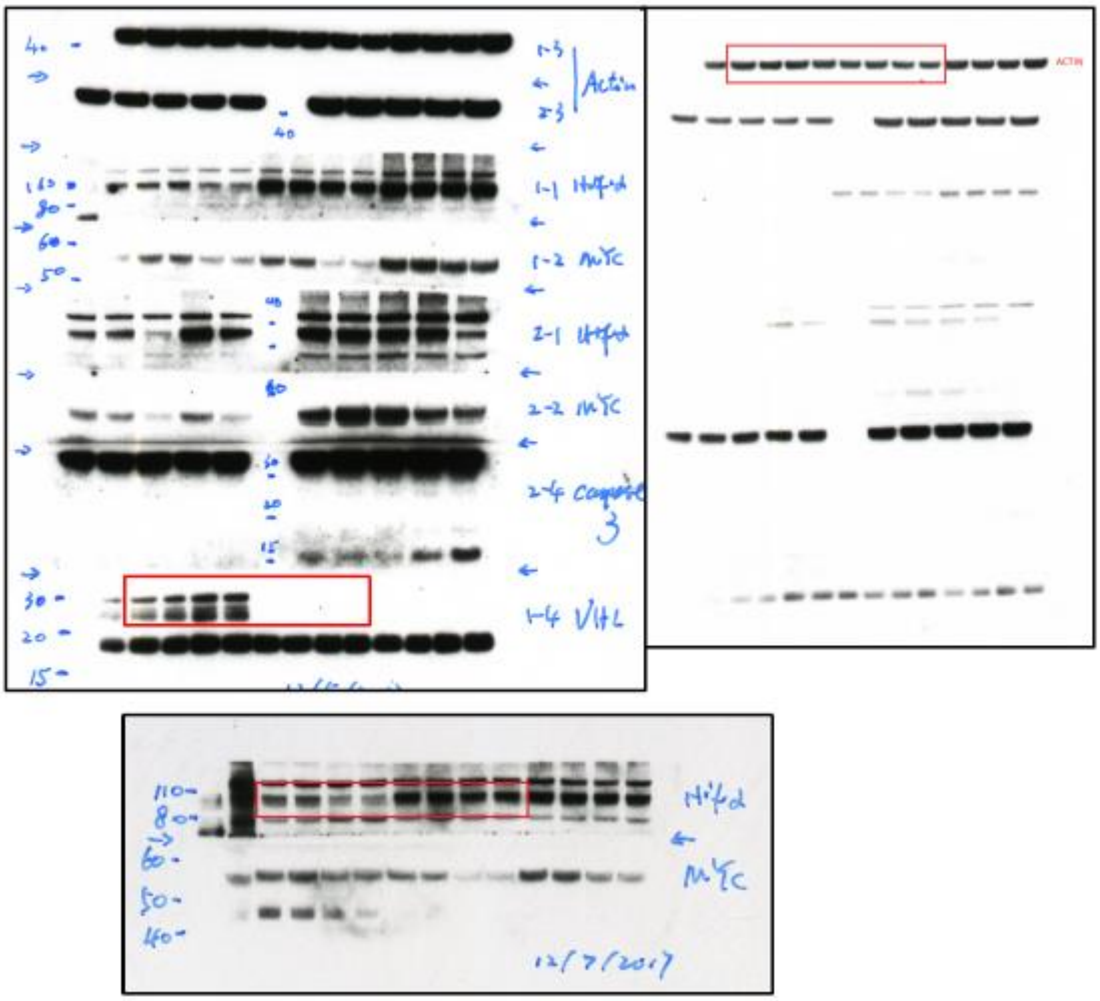
Actin
MYC
VHL
ACTIN
MYC
12/7/2017

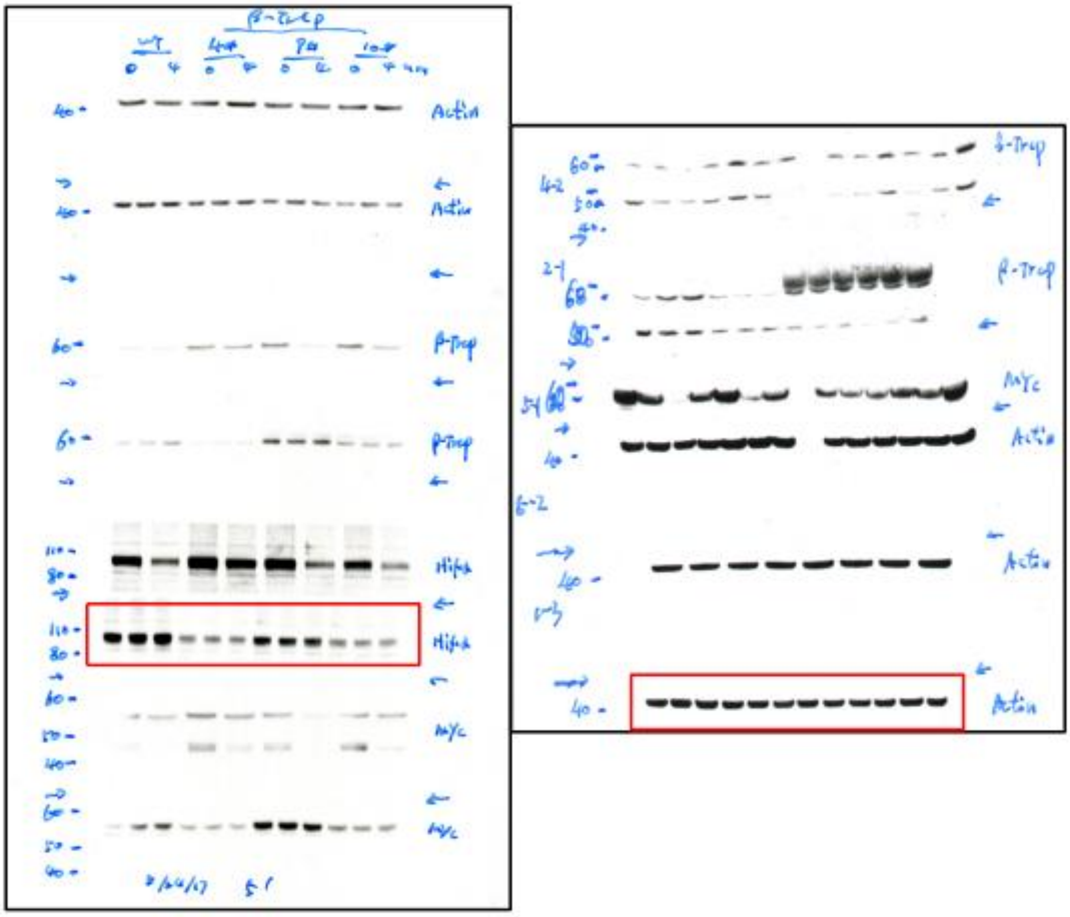
β-Trcp
Actin
β-Trcp
Myc
Actin

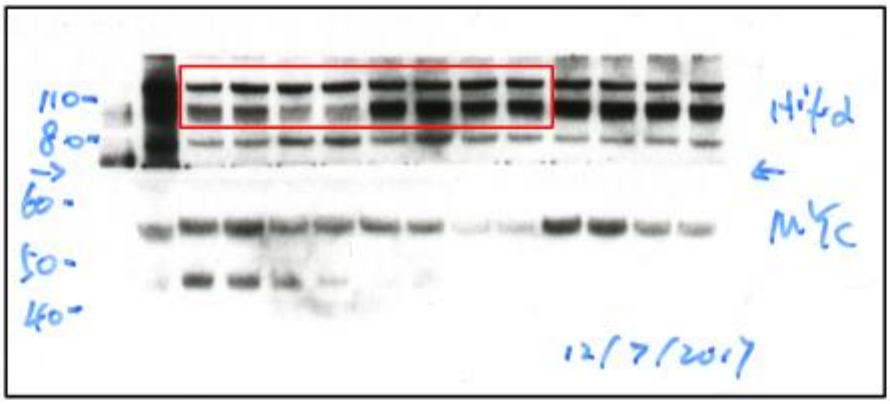
110
80
60
50
40
MYC
12/7/2017

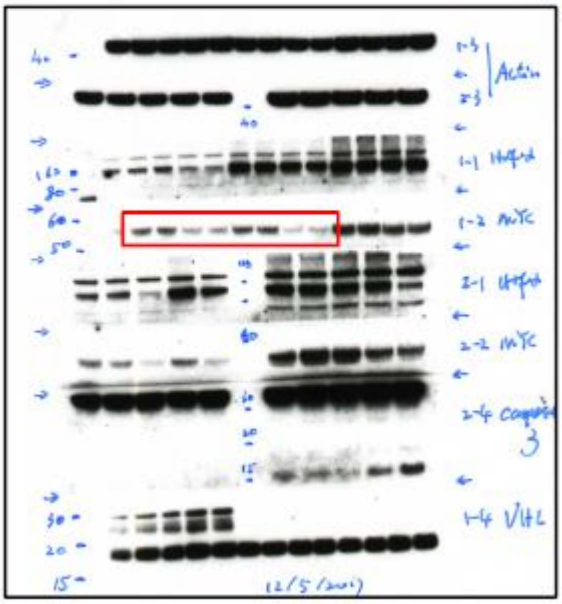
Actin
1-4 VHL
12/5/2017

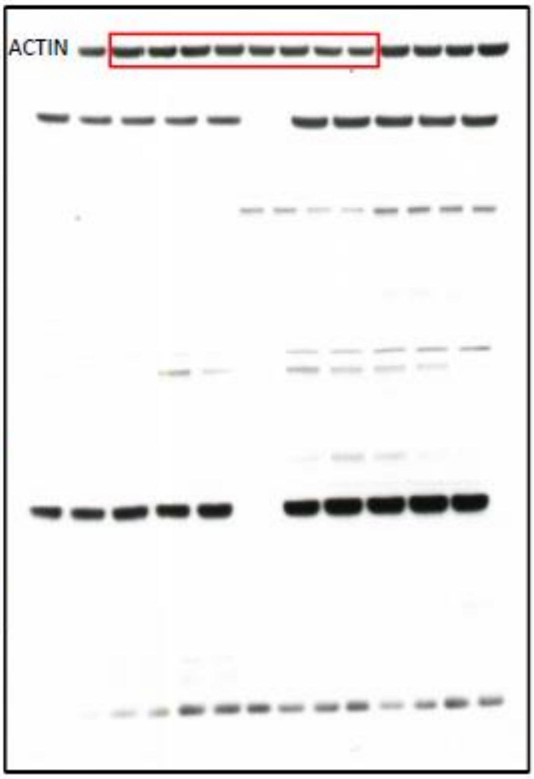
ACTIN

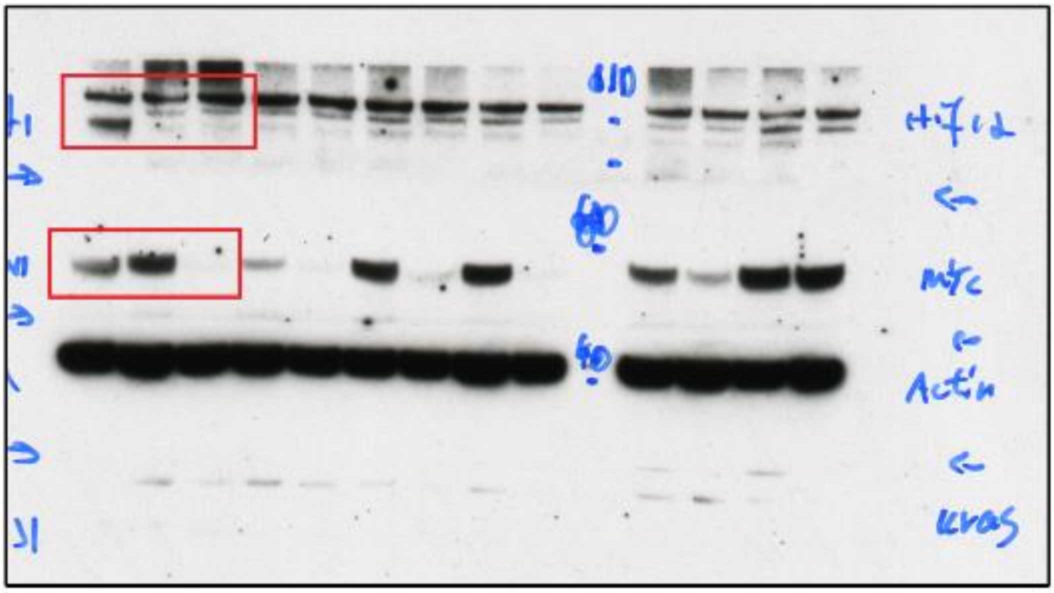
Hif1a
myc
Actin
kras

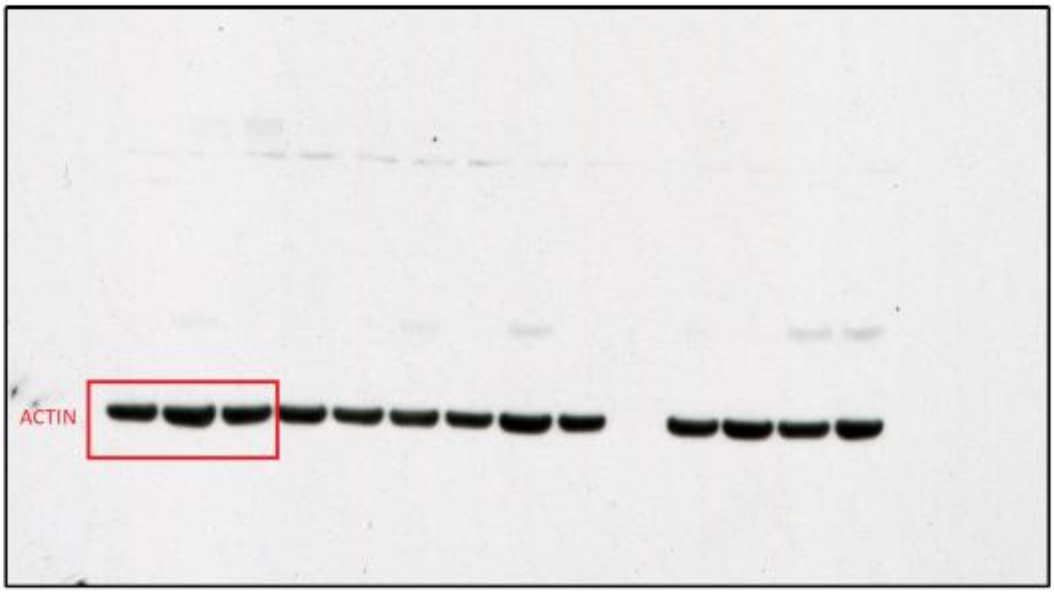
ACTIN

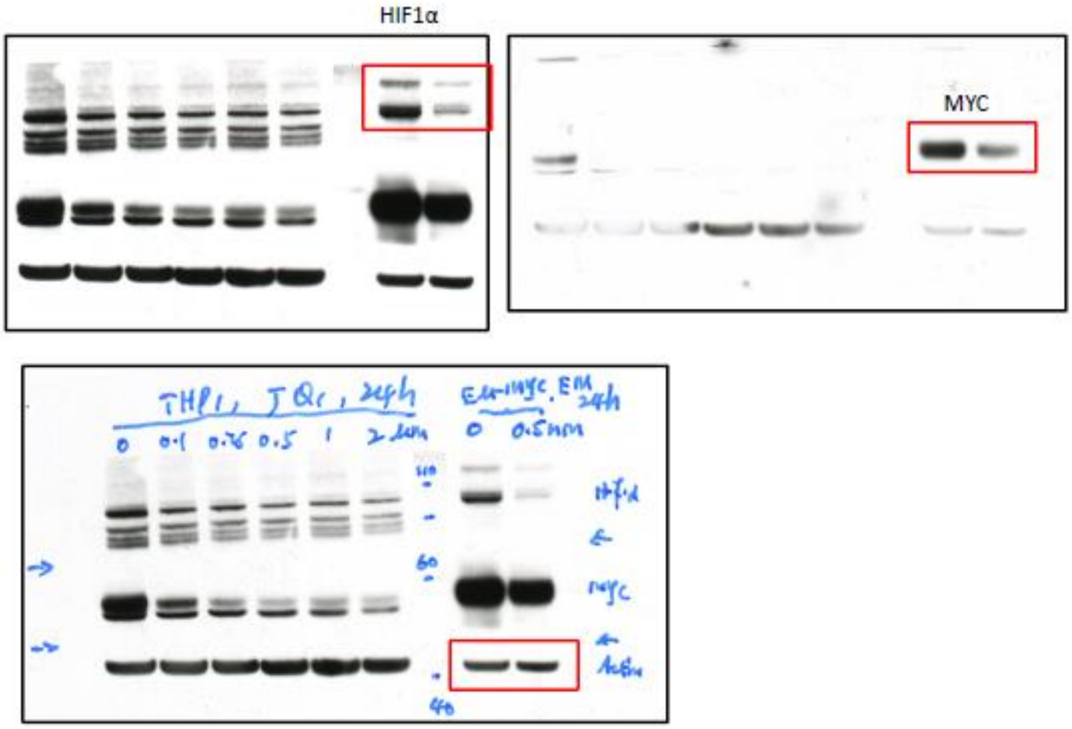

Figure S14. The uncropped Western blots.
